# Advances and Challenges of Capacitive Micromachined Ultrasonic Transducers in Medical Imaging

**DOI:** 10.3390/mi17040486

**Published:** 2026-04-16

**Authors:** Yuanyu Yu, Xin Liu, Jiujiang Wang, Shuang Zhang

**Affiliations:** 1 School of Artificial Intelligence, Neijiang Normal University, Neijiang 641100, China; cdyu@163.com (Y.Y.); zhang.s@njtc.edu.cn (S.Z.); 2 School of Mathematics and Computer Science, Northwest Minzu University, Lanzhou 730030, China; xinliu2024@xbmu.edu.cn

**Keywords:** capacitive micromachined ultrasonic transducers (CMUTs), CMUTs fabrication process, ultrasound imaging, photoacoustic imaging

## Abstract

Capacitive micromachined ultrasonic transducers (CMUTs) have been developed over the past 30 years and achieved practical applications in both medical imaging and industrial non-destructive testing. This article presents the fundamental principles of CMUTs and surveys fabrication technologies, offering a comprehensive review of major advances and challenges in medical ultrasound and photoacoustic imaging applications. The article further reviews and analyzes three primary challenges currently confronting CMUTs in medical imaging applications: lower output acoustic pressure, dielectric charging effects, and the need for high bias voltage. It also presents and discusses a potential combined approach to comprehensively address these challenges, with the aim of enhancing CMUT performance and broadening clinical adoption.

## 1. Introduction

Ultrasound technology plays an extremely important role in the biomedical field because of its advantages, including non-invasiveness, absense of ionizing radiation, real-time imaging, and high soft tissue contrast. It is widely used in various fields, including disease diagnosis [1], treatment [2], and neuromodulation [3]. In an ultrasound system, the ultrasonic transducer is the key component that converts ultrasound waves into electrical signals, and vice versa. In biomedical applications, based on their structures and working principles, ultrasonic transducers are mainly divided into piezoelectric transducers [4] and micromachined ultrasonic transducers (MUTs) [5]. Piezoelectric transducers primarily use materials such as lead zirconate titanate (PZT) [6], lead magnesium niobate–lead titanate (PMN-PT) [7], potassium sodium niobate (KNN) [8], PZT single crystals [9] and polyvinylidene fluoride (PVDF) [10]. The function of piezoelectric transducers is based on the piezoelectric effect, whereby the materials deform mechanically when an electric field is applied and, conversely, produce electrical signals in response to incoming ultrasonic waves. Because of their high efficiency, excellent sensitivity, and stability, piezoelectric transducers are widely utilized in biomedical ultrasound imaging applications. MUTs are ultrasonic devices fabricated using microelectromechanical systems (MEMS) technology. Their fundamental principle lies in efficiently converting electrical energy into mechanical vibrations and vice versa, enabling the transmission and reception of ultrasound waves. MUTs represent a significant advancement in ultrasonic technology, enabling the miniaturization and enhanced flexibility of ultrasonic transducers for biomedical applications. Based on their operating principles, MUTs are mainly divided into two types: CMUTs [11] and piezoelectric micromachined ultrasonic transducers (PMUTs) [12]. CMUTs operate based on electrostatic forces by applying a bias voltage between the vibrating membrane and the fixed electrode to generate an electrostatic field. The vibration of the membrane is then used to transmit and receive ultrasound waves. PMUTs operate based on the piezoelectric effect. By applying an electric field to the piezoelectric thin film, the membrane is driven to vibrate and generate ultrasound waves, or conversely, the membrane receives ultrasound waves and converts them into electrical signals. Compared to PMUTs, CMUTs offer a wider frequency response range, particularly excelling at higher frequencies [13]. This makes them more suitable for biomedical applications requiring high-resolution and high-frequency ultrasound, such as medical ultrasound imaging and various ultrasound instruments. In this article, the advances and challenges of CMUTs in medical imaging are comprehensively reviewed.

This article is organized as follows. Section 2 introduces the fundamentals of CMUTs. Section 3 focuses on the advances in medical ultrasound imaging applications of CMUTs. Section 4 reviews the advanced applications of CMUTs in photoacoustic imaging. Section 5 reviews the three main challenges associated with CMUTs in medical ultrasound imaging. Section 6 explores the practical advantages of CMUT in medical diagnostics, the interaction between medical imaging and its inherent challenges, as well as the interconnections among various challenges.

## 2. Fundamentals of CMUTs

### 2.1. Structure and Principle of CMUTs

**Figure 1:**
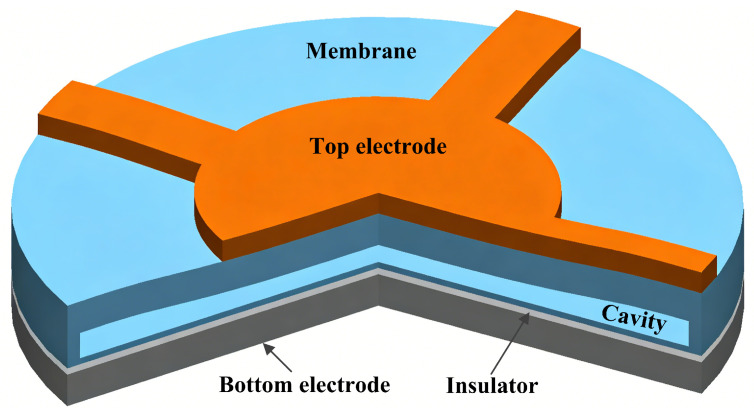
Structure of a CMUT cell.

A conventional CMUT cell typically consists of a thin, movable vibrating membrane suspended above a fixed electrode, with a small vacuum cavity between them, constituting a miniature parallel-plate capacitor. Figure 1 shows the structure of a CMUT cell fabricated by the MEMS process. The vibrating membrane is typically made of materials such as silicon nitride, silicon or other thin-film materials. The thickness, shape, and area of the membrane are key parameters that determine the performance of a CMUT. In general, CMUTs are fabricated on a silicon wafer, which also acts as the substrate. The fixed bottom electrode may be realized with a highly doped silicon wafer or a deposited metal layer. An insulator is generally deposited above the bottom electrode. A vacuum cavity is designed between the insulator and the vibrating membrane to provide a space for the membrane to vibrate. The metal top electrode is patterned on the membrane. Therefore, the CMUT cell can be regarded as a capacitor with a varying gap.

**Figure 2:**
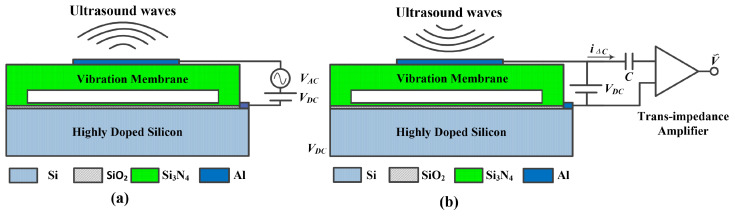
Working principle of CMUTs. (**a**) Transmit mode. (**b**) Receive mode.

The CMUT cell is a capacitive device that operates based on electrostatic principles. When a DC bias voltage is applied between the top electrode and the fixed bottom electrode, the electrostatic force attracts the membrane toward the substrate, causing it to deflect downward. This deflection determines the operating point of the CMUT, which is critical for optimizing electromechanical coupling efficiency, sensitivity, and ultrasonic output performance. Figure 2 shows the working principle of CMUTs in transmit and receive modes, respectively. In both modes, a DC bias voltage should be applied across the electrodes to maintain the operating point of the CMUT. In transmit mode, pulses or AC signals are superimposed on the DC bias voltage to generate varying electric fields that drive the membrane vibration and generate ultrasound waves, as illustrated in Figure 2a. The ultrasound waves generated by the vibration of the membrane are determined by the resonant frequency of a CMUT.

The resonant frequency of a CMUT cell is determined by the material and dimensions of the membrane. For a circular CMUT, its resonant frequency can be approximated using the formula for calculating the fundamental resonant frequency of a clamped circular plate in air as (1).

(1) $$f_{plate}=0.47\frac{h}{r^{2}}\sqrt{\frac{E}{\rho (1-\sigma^{2})}}$$

where *h* denotes the membrane thickness, *r* is the membrane radius, *E* represents the Young’s modulus of the material of the membrane, ρ indicates the material density, and σ stands for the material’s Poisson’s ratio. In practical medical applications, the CMUT operates in an immersed environment. Consequently, the liquid’s viscosity reduces the resonant frequency of the CMUT. For an immersed circular CMUT, assuming the membrane dimensions are much smaller than the acoustic wavelength, the damped resonant frequency $f_{r}$ can be determined using the following Equation (2) [14].

(2) $$f_{r}=\frac{\frac{2.98h}{r^{2}}\sqrt{\frac{E}{\rho_{p}\left(1-\sigma^{2}\right)}}}{\sqrt{1+0.67\frac{\rho_{l}r}{\rho_{p}h}}}$$

In this equation, $f_{r}$ denotes the damped angular resonant frequency of the circular membrane, $\rho_{p}$ and $\rho_{l}$ correspond to the densities of the membrane and the surrounding liquid, respectively. This demonstrates that both the properties and the dimensions of the membrane determine the center frequency of a CMUT cell.

In receive mode, incident ultrasound waves cause the CMUT membrane to vibrate, resulting in a change of capacitance between the electrodes. Under the influence of a DC bias voltage, the changing charge between the CMUT electrodes is output as a weak current signal $i_{\Delta C}$, which is then converted into a voltage signal through a trans-impedance amplifier circuit, as demonstrated in Figure 2b.

(3) $$i_{\Delta C}\left(t\right)=V_{DC}\frac{dC_{CMUT}\left(t\right)}{dt}=V_{DC}\;\cdot \;\varepsilon A\frac{d}{dt}\frac{1}{g-\omega (t)}$$

where *A* is the surface area of the top electrode, and *g* is the gap height. The incident ultrasonic wave impacts the vibrating membrane of the CMUT, causing a displacement ω(t) that varies over time *t*, which in turn changes the capacitance of the CMUT cell $\frac{dC_{CMUT}\left(t\right)}{dt}$. Under the action of the DC bias voltage $V_{DC}$ applied to the CMUT electrodes, the varying capacitance is converted into a current signal. As shown in Equation (3), the current signal $i_{\Delta C}\left(t\right)$ is directly related to the displacement ω(t) of the vibrating membrane, where ω(t) reflects the characteristics of the ultrasound wave applied to the CMUT membrane. Therefore, by converting $i_{\Delta C}\left(t\right)$ into voltage signals, the incident ultrasonic waves can be reconstructed.

### 2.2. Fabrication of CMUTs

CMUTs are MEMS devices, and their fabrication processes can mainly be divided into two categories: surface micromachining [15] and wafer bonding [16]. Early CMUTs were primarily fabricated with surface micromachining technology. In recent years, an increasing number of CMUTs have been fabricated using the wafer-bonding process.

#### 2.2.1. Surface Micromachining Process

**Figure 3:**
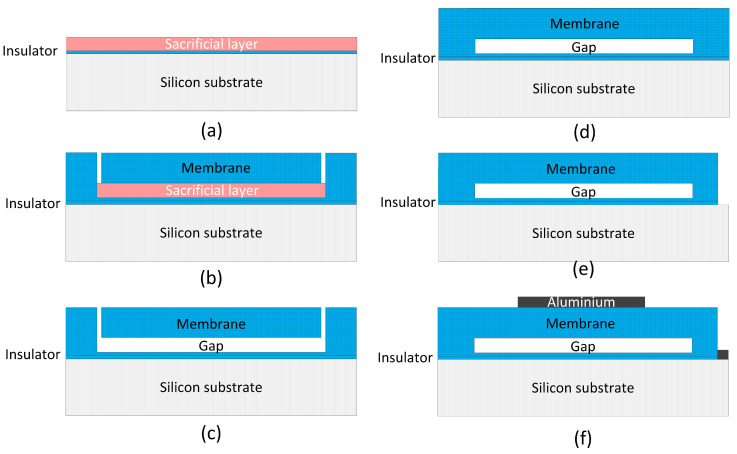
Typical fabrication process of surface micromachining techniques. (**a**) Sacrificial layer deposition; (**b**) etching pathways to the sacrificial layer; (**c**) creating the gap; (**d**) sealed vacuum gap; (**e**) bottom electrode area exposed; (**f**) pattern electrodes and connections.

A typical surface micromachining fabrication process is described in Figure 3 [17]. Firstly, an insulator layer is deposited on a highly doped silicon wafer, which acts as the common bottom electrode. A sacrificial layer is also deposited above the insulator layer, shown in Figure 3a. Then the sacrificial layer is patterned to define the shape of cavities. Subsequently, a structure layer acting as the membrane and support layer is coated above the whole wafer. To create etching pathways to the sacrificial layer, release holes are made outside the membrane to establish channels for chemical etching, as shown in Figure 3b. The sacrificial layer between the membrane and the insulator is completely removed by wet etching through the release holes, thereby creating the gap, as shown in Figure 3c. A vacuum gap is formed by sealing the etch holes in a low-pressure chamber, shown in Figure 3d. Subsequently, the non-conductive material covering the bottom electrode pads is removed via etching to expose the silicon substrate, as described in Figure 3e. Finally, a metal layer is coated and then patterned as the electrode pads and connections, as shown in Figure 3f.

In addition to the traditional process, Coppa et al. developed a reverse fabrication process, starting with a silicon nitride film to construct CMUT structures without etch holes [18]. PolyMUMPs (polysilicon multi-user MEMS processes) from MEMSCAP (MEMSCAP S.A., Crolles, France) is a standardized surface micromachining process platform that is also used in the fabrication of CMUTs. Tawfik et al. developed surface micromachined CMUTs with PolyMUMPs technology [19]. This device features a design that reduces the gap, enhancing the output pressure and receiving sensitivity of the CMUTs while also lowering the operating bias voltage.

As traditional silicon-based CMUTs lack sufficient flexibility, which limits their applications in wearable ultrasound patches and conformal ultrasound imaging. In recent years, polymer-based CMUTs (polyCMUTs) have been developed. This approach uses polymer structures in place of silicon-based components, enabling flexible transducers and cost savings in materials and fabrication. Pang et al. introduced a flexible CMUT fabrication method based on a surface micromachining process [20]. In this approach, they used polyethylene terephthalate (PET) as the substrate, photosensitive polymer SU-8 as the sidewalls and vibrating membranes, and copper as the sacrificial layer. Gerardo et al. proposed a method for fabricating polyCMUTs using SU-8 and Omnicoat, in which SU-8 served as both the structure and membrane, and concentrated Omnicoat acted as the sacrificial layer. They fabricated a 64-element polyCMUT array and conducted imaging experiments [21]. Lucarini et al. demonstrated a surface micromachining fabrication process for flexible polyCMUTs, using polyimide (PI) as both the substrate and membrane, with aluminum as the sacrificial layer, which enables bending of the devices to very small curvature radii down to 1 mm [22]. Omidvar et al. introduced 1D and 2D flexible polyCMUTs for conformal ultrasonography, with the 1D array containing 32 elements [23]. The polyCMUTs employ PI as the substrate, SU-8 as both the membrane and sidewalls, and lift-off resist as the sacrificial layer, with fabrication achieved by surface micromachining. The curvature radius reached 3 cm, and the acoustic responses were relatively consistent across different bending states. Subsequently, Omidvar et al. used the same process to fabricate a 128-element flexible polyCMUT with a length of 91 mm and conducted imaging experiments of steel plates, wires, and biological tissues under different curvatures [24].

#### 2.2.2. Wafer-Bonding Process

**Figure 4:**
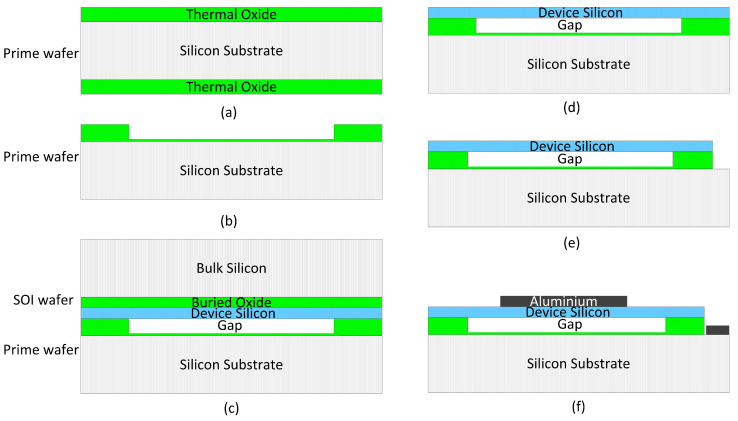
Typical process of wafer-bonding techniques. (**a**) Thermal oxidation on the prime wafer; (**b**) oxide etch to create the cavity; (**c**) wafers are bonded to seal the gap; (**d**) remove bulk silicon and buried oxide layer; (**e**) bottom electrode area exposed; (**f**) pattern electrodes and connections.

The wafer-bonding process for CMUTs was developed by Stanford University in the early 2000s [16]. The primary characteristic of this technique is that the CMUT membrane and cavity are fabricated on two distinct wafers, which are subsequently bonded together. This separate fabrication of the cavity and membrane allows for more precise and flexible control over their dimensions compared to the surface micromachining process. Additionally, this method is less complex than the sacrificial release approach, as it eliminates the need for creating sacrificial channels and holes. The fabrication process of CMUTs using the wafer-bonding technique is illustrated in Figure 4. In this approach, the device’s cavity and substrate are prepared on a prime silicon wafer, while the membrane using formed with a silicon-on-insulator (SOI) wafer. The process begins with the deposition of a thin thermal oxide layer onto a low-resistivity prime silicon wafer, as in Figure 4a. The cavity is then created in the silicon dioxide layer, and the unwanted thermal oxide layer at the bottom of the substrate is removed, as depicted in Figure 4b. Subsequently, the prime silicon wafer and the SOI wafer are bonded together in a vacuum environment to ensure that the cavity is vacuum-sealed, as shown in Figure 4c. Since the membrane is formed from the device silicon layer of the SOI wafer, the bulk silicon and buried oxide layer in the SOI wafer are etched away to form the membrane structure, as described in Figure 4d. The silicon layer outside the membrane and the oxide layer are further etched to create a pathway through the top layer to the low-resistivity substrate acting as the bottom electrode, as shown in Figure 4e. Finally, aluminum is sputtered and patterned to form the electrodes and connections, shown in Figure 4f.

The use of silicon direct bonding for CMUT fabrication has significantly enhanced the large-scale manufacturing capabilities and performance consistency of the devices. However, the cost of SOI wafers is relatively high, and the uniformity of the film thickness remains constrained. To solve this problem, Logan et al. proposed a bonding solution using a fully silicon nitride film, employing a user-grown silicon nitride layer as the membrane. This approach not only reduces costs but also optimizes the mechanical properties [25]. Chen et al. developed an oxide-based wafer-bonding process that eliminates the dependence on SOI wafers, simplifying the procedure and enhancing the uniformity of the film [26]. To enhance device reliability and performance, various improved bonding processes have been developed. Park et al. [27] fabricated CMUTs with an improved insulation layer structure through a series of continuous thermal oxidation steps, localized oxidation of silicon (LOCOS), and direct wafer bonding. This approach strengthens the device’s insulation capability, effectively reduces leakage, and increases the breakdown voltage. To address the thermal stress issues that may arise from high-temperature bonding, some studies have focused on developing low-temperature bonding processes. Zhao et al. [28] achieved direct bonding at temperatures below 350 °C, significantly reducing thermal deformation. This advancement not only minimizes the impact of thermal stress on the device structure but also enhances the overall reliability and performance of the bonded materials. Li et al. [29] utilized polymers such as benzocyclobutene (BCB) as adhesives to complete bonding at a low temperature of 250 °C. This temperature is fully compatible with back-end complementary metal-oxide semiconductor (CMOS) processes, providing a critical pathway for the monolithic integration of CMUTs with analog front-end circuits. Zhang et al. studied the wafer-bonding process using SU-8 as an adhesive layer, employing glass and transparent electrodes, which allows CMUTs to exhibit optical transparency [30]. Similarly, Yildiz et al. [31] used low-temperature co-fired ceramics (LTCC) that are capable of anodic bonding to achieve the stable fabrication of CMUTs with varying membrane radii. This method allows for precise control over the device structure and ensures reliability, making it suitable for applications requiring diverse membrane geometries while maintaining consistent performance.

#### 2.2.3. Comparison of the Fabrication Process

Surface micromachining technology and wafer-bonding technology each have distinct advantages and disadvantages in the fabrication of CMUTs. These differences directly influence the performance, cost, integration level, and final applications of CMUTs. Table 1 summarizes the comparison of the conventional surface micromachining process and the wafer-bonding process of CMUTs.

In the conventional surface micromachining process, the need for sacrificial layer release and the deposition of multilayer films creates limitations on the dimensions of CMUT cavities and membranes to avoid issues such as membrane breaking and stiction during the wet release of the sacrificial layer [15]. In wet etching, the membrane is prone to rupture if the membrane residual stress is too high, because the etchant removes the underlying sacrificial material, leaving the membrane unsupported and subjected to the forces generated by residual-stress gradients. Furthermore, the membrane thickness must exceed a critical value to avoid stiction. This problem occurs during the drying process after wet sacrificial layer removal, as evaporating liquid generates capillary forces from surface tension that pull the membrane toward the substrate. If the membrane lacks sufficient stiffness, these forces overcome its elastic restoring force, resulting in irreversible adhesion and device failure [32]. It is also a challenge to achieve small gap heights for the cavities. Therefore, the structural flexibility of this fabrication process is restricted. The membrane of CMUTs is formed by film deposition on the wafer, which includes techniques such as low-pressure chemical vapor deposition (LPCVD) or plasma-enhanced chemical vapor deposition (PECVD). These film deposition methods have issues with uniformity. Even on the same wafer, the membrane thickness of CMUTs at different locations can vary [33]. As a result, the uniformity of the membranes produced by these methods is generally not sufficient. In addition, the vacuum cavities of CMUTs are made by depositing films in an LPCVD/PECVD chamber; the operating gas pressure inside the chemical vapor deposition (CVD) chamber and potential sealing failures may lead to inadequate vacuum conditions in the CMUT cavities. Furthermore, the fill factor of the CMUT array is fundamentally constrained by the necessary sacrificial release channels and etch holes, which occupy valuable active area. This situation is more significant for high-frequency, high-performance CMUT arrays. Therefore, the frequency range of CMUTs fabricated by surface micromachining is moderate. However, the surface micromachining process utilizes conventional MEMS fabrication equipment and demonstrates good compatibility with integrated circuit manufacturing techniques. This compatibility facilitates the easier integration of CMUTs with CMOS circuits, enabling the creation of compact and high-performance ultrasound systems [34].

The wafer-bonding process allows for the independent fabrication of CMUT membranes and substrate wafers, followed by bonding, which provides greater flexibility in device structure design. This process enables the creation of complex substrate structures or combinations of different materials. For instance, transparent CMUTs can be achieved by bonding indium tin oxide (ITO)-coated glass wafers with SU-8 or BCB as the adhesive layer, resulting in optical transparency [30,35]. The wafer-bonding process enables whole wafer bonding in a low-pressure condition, which is crucial for enhancing the vacuum cavity sealing and the stability of CMUT devices [36]. Since the cavity is defined by another wafer, it allows for the realization of a very small and uniform gap, significantly reducing the collapse voltage. The vibrating membranes of CMUTs are provided by the SOI wafer, eliminating the need for a sacrificial layer release during the fabrication process. This results in a smaller cell pitch, increased fill factor [29], and a larger effective working surface area within CMUT arrays, making it suitable for high-frequency applications. The drawbacks of the wafer-bonding process include the need for bonding equipment during fabrication, and if SOI wafers are used, the costs may be relatively high. The wafer-bonding process has high requirements for the surface flatness, cleanliness, and alignment accuracy of the bonded surfaces. During bonding, mismatched thermal expansion coefficients between different materials may introduce residual stress, which can affect the flatness of the membrane and the performance of the device [37].

As a conclusion, the core differences between surface micromachining and wafer-bonding methods in CMUT fabrication can be summarized as follows. Surface micromachining utilizes conventional MEMS equipment to deposit and etch thin film layers layer by layer on a wafer. This process involves a relatively high number of steps and masks and is constrained by factors such as membrane stress, poor gap control, and low fill factors due to release holes. Nonetheless, it features lower manufacturing costs and flexibility, making it suitable for low-to-medium performance applications, such as small-batch fabrication in laboratories. In contrast, wafer-bonding typically requires a bonding machine and SOI wafers, involving a hybrid process where two independently processed wafers are bonded together. This approach features fewer process steps and masks, delivering better uniformity, better gap control, and vacuum sealing. However, it entails higher costs and more stringent process controls, making it ideal for high-performance applications used in medical imaging and industrial mass production.

### 2.3. Comparison of CMUTs with Piezoelectric Transducers

CMUTs and traditional piezoelectric ultrasound transducers have fundamental differences in working principles, material systems, manufacturing processes, and system integration capabilities. Therefore, there are key differences between them, as listed in Table 2.

CMUTs exhibit significant advantages in key performance metrics. CMUTs have a relatively wider bandwidth, typically exceeding 100%, which is substantially higher than that of PZT ultrasonic transducers, which are generally no more than 80%. This advantage makes CMUTs particularly suitable for advanced imaging modes such as harmonic imaging, broadband pulse-echo imaging, and multi-frequency operations. Additionally, the center frequency of CMUTs is determined by the material and dimension of the membrane; therefore, they offer greater central frequency tunability and array density, enabling the construction of high-density 2D arrays. CMUTs also have lower acoustic impedance (approximately 2–3 MRayl), which is closer to that of human soft tissue (1.5–1.6 MRayl) [38]. In contrast, the acoustic impedance of PZT (approximately 30 MRayl) is much higher than that of human tissue. Therefore, to improve transmission efficiency at the interface between PZT and human tissue, matching layers should be utilized.

The primary advantage of CMUTs is high compatibility with CMOS processes. CMUT arrays can be batch-fabricated on silicon substrates, resulting in a high level of device consistency. More importantly, CMUT can achieve monolithic or heterogeneous integration with analog front-end (AFE) circuits, significantly reducing the interconnection pathway and thereby effectively reduce parasitic capacitance and noise interference, enhancing the signal-to-noise ratio (SNR). In contrast, PZT is limited by the processing characteristics of ceramic materials and typically requires wire bonding to connect separate electronic components, which restricts the miniaturization of probes and poses challenges.

CMUTs operate based on the principle of electrostatic force and exhibit an inherent nonlinear relationship between the driving voltage and the output acoustic pressure [17]. In comparison, piezoelectric transducers rely on the piezoelectric effect, exhibiting an approximately linear strain-electric field relationship. Thus, CMUTs offer moderate linearity, which can be enhanced through reasonable bias voltage control, structural optimization, and back-end signal processing. Piezoelectric transducers tend to experience significant self-heating due to dielectric and mechanical losses. In contrast, CMUT energy conversion occurs mainly through capacitance variation and membrane vibration, resulting in low internal losses and no need for acoustic impedance matching layers, thereby minimizing self-heating [39,40]. Regarding thermal performance, piezoelectric materials lose their effect above the Curie temperature, leading to poor thermal stability. CMUTs have no such limitation. CMUTs are generally fabricated on silicon processes with higher thermal conductivity than PZT, enabling rapid heat conduction and radiation, thereby providing superior thermal stability. These advantages make CMUTs highly suitable for high-density arrays integrated with CMOS. In terms of biocompatibility, traditional piezoelectric materials like PZT contain lead, requiring encapsulation to prevent trace ion release despite low dissolution rates after sintering. CMUTs, typically made from silicon-based materials, demonstrate good biocompatibility.

However, traditional piezoelectric transducers still hold advantages in terms of output pressure and penetration depth, particularly in therapeutic applications such as high-intensity focused ultrasound (HIFU), where they demonstrate more stable and reliable performance. Additionally, CMUTs generally need a relatively high DC bias voltage in both transmission and receive modes, which raises safety considerations in biomedical applications [41].

## 3. Applications of CMUTs in Ultrasound Imaging

Compared to traditional PZT transducers, CMUTs offer wider bandwidth, higher integration, and greater design flexibility. They have been extensively studied and applied in conventional medical ultrasound imaging. Several manufacturers now offer commercial CMUT-based probes or systems, which are used successfully in various clinical fields.

### 3.1. Intravascular Ultrasound Imaging

Intravascular ultrasound (IVUS) imaging is the most commonly used interventional imaging technique for assessing coronary arteries [42]. In the IVUS imaging process, a catheter equipped with a miniaturized ultrasound transducer is advanced along a guidewire to the distal end of the target lesion. High-resolution images of the vascular structure are generated by utilizing the reflected ultrasound signals. The wideband and high-frequency characteristics of CMUTs make them suitable for echocardiography and flow imaging. Additionally, their miniaturization capability makes them highly suitable for IVUS, providing high-resolution images of the vascular wall [43]. Phased CMUT arrays and dual-ring arrays are designed in IVUS imaging [44].

Zangabad et al. achieved real-time IVUS imaging at a frame rate of 20-frame/s using coded excitation and CMUT phased arrays [45]. The CMUT array exhibits a 6 dB bandwidth of 25 MHz and a center frequency of 20 MHz under a 30 V DC bias. Imaging was conducted on a linear model and human coronary artery plaques. The axial resolutions for short pulse and coded excitation imaging are 60 μm and 70 μm, respectively, while the lateral resolutions are 270 μm and 245 μm, respectively. Gurun et al. developed a 1.4 mm diameter dual-ring CMUT array using CMUT-on-CMOS technology for real-time IVUS and intracardiac echocardiography (ICE) imaging [46]. The dual-ring array consists of 56 transmitting elements and 48 receiving elements, arranged on two independent concentric rings. The center frequency of the CMUTs is 20.1 MHz, with a fractional bandwidth of 43%. The system was tested on a wire model and ex vivo chicken heart samples, achieving axial and lateral point resolutions of 92 μm and 251 μm, respectively. Pekař et al. [47] developed a tunable frequency, 32-element, 1D phased-array CMUT for IVUS imaging. The CMUTs are operating in collapse mode, in which the DC bias voltage is above the pull-in voltage. By adjusting the DC bias voltage, transmitting pulse frequency, and the number of transmitting pulses, three modes of penetration mode, resolution mode, and general imaging mode were established. In the resolution mode, the axial resolution achieved was 55 μm, while the lateral resolution was 0.035 rad, with a penetration depth of 16 mm. Table 3 summarizes the research of CMUTs in IVUS imaging.

These studies demonstrate the potential of CMUTs for miniaturizing IVUS catheters and achieving high-resolution imaging. For IVUS applications that require miniaturized probes, the advantages of CMUT become even more apparent due to its compatibility with circuit integration. Tekes et al. designed a 20 MHz single-chip dual-ring array for IVUS that features both forward-looking and side-looking capabilities [48]. The array consists of 56 transmitting elements and 48 receiving elements, which are fabricated directly on the processed CMOS wafer using CMUT-on-CMOS technology. Lim et al. [49] developed a 40 MHz CMUT array interface system on chip (SoC) for guidewire IVUS imaging, which operates with only two wires for power supply and image data transmission. This system demonstrates the overall functionality of the IVUS imaging system and achieves wireless transmission of IVUS images using a 12-element CMUT array. Zhang et al. designed a highly integrated SoC for IVUS that employs plane wave transmit beamforming at 40 MHz for IVUS on guidewires or microcatheters [50]. The front-end circuit consists of a 20-channel ultrasound transmitter and receiver array, which is connected to a CMUT array. In transmission, all 20 transmitter channels are simultaneously stimulated with the continuously adjustable analog delay, with the unit delay controlled by a voltage-controlled delay line, thereby generating a controllable plane wave within a range of ±50°.

**Table 3:** CMUTs used in IVUS imaging.

Array	Frequency	Bandwidth	Axial Resolution	Lateral Resolutions
Dual-ring array [46]	20.1 MHz	43%	92 μm	251 μm
Ring array [51]	19 MHz	69%	78 μm	0.051 rad
Phased array [45]	20 MHz	125%	60/70 μm	270/245 μm
Phased array [47]	20.8 MHz	56–74%	55 μm	0.035 rad
Linear array [52]	35.6 MHz	28.5%	N/A *	277 μm
Linear array [53]	20 MHz	100%	500 μm	N/A
Linear array [54]	9.2 MHz	96%	250 μm	N/A
Linear array [55]	15 MHz	100%	N/A	N/A

* Not available.

Although CMUTs have shown considerable potential for IVUS in various experimental studies, traditional piezoelectric ultrasonic transducers remain the mainstream in IVUS applications to this day. This is primarily attributable to their demonstrated long-term reliability in clinical settings, well-established mature manufacturing processes, and decades of extensive clinical validation and regulatory approval. Traditional piezoelectric ultrasonic transducers can provide stable performance, sufficient penetration depth, and robust integration within miniature catheters. The main obstacles to promoting CMUTs in IVUS include high bias voltage demands that raise electrical safety concerns, relatively lower output pressure and loop gain in miniaturized designs, and difficulties in long-term stability under vascular conditions. These factors maintain the dominance of piezoelectric technology in current clinical practice.

### 3.2. General Ultrasound Imaging

In the general non-invasive ultrasound imaging applications, most studies use commercial CMUT probes. In 2009, Hitachi Medical Corporation (Tokyo, Japan) announced the launch of the first commercial CMUT product, Mappie, which was a linear array probe specifically designed for breast imaging [56]. In 2020, the fourth-generation CMUT (4G CMUT) model of SML44 was launched by Hitachi for tissue harmonic imaging, color Doppler flow imaging, and tissue elastography [57]. In 2018, Butterfly Network (Butterfly Network, Inc., Guilford, CT, USA) launched the handheld medical imaging system, Butterfly iQ [58]. This FDA-approved system features a 2D CMUT array with 9000 elements, achieving a high level of portability for ultrasound devices [59]. In 2020, the second-generation product, Butterfly iQ+, was launched with enhancements in frame rates, optimized beamforming, and power efficiency [60]. In 2024, Butterfly Network introduced the third-generation clinical ultrasound probe, Butterfly iQ3, with improved processing power and a smaller probe face. It was awarded the Best Medical Technology at the 2024 Prix Galien USA Awards [61]. The frequency range of Butterfly iQ3 is 1 MHz–12 MHz. Kolo Medical (Kolo Medical, Inc., Suzhou, China) developed linear and matrix ultrasound probes based on CMUTs. The linear transducer, L38-22, is used for superficial imaging with a center frequency of 30 MHz. In addition to the manufacturers mentioned above, Vermon (Vermon, Torus, France) and ACULAB (Acusto-Electronics Laboratory, Rome, Italy) also provide commercial CMUT probes, which are utilized in experimental research [62]. Currently, at least seven companies have launched 19 types of CMUT-based ultrasound products, of which four have received clinical approval [63].

The Butterfly iQ series products have demonstrated significant advantages in multiple general ultrasound imaging fields because of their portability and versatility. Table 4 lists the features of Butterfly iQ, iQ+ and iQ3.

#### 3.2.1. Cardiovascular Ultrasound Imaging

Jujo et al. [64] proposed and validated the feasibility of a point-of-care ultrasound (POCUS) training course for medical students using the handheld ultrasound of Butterfly iQ. This work provides valuable insights and directions for improvement for future large-scale studies. Karimpour et al. used the Butterfly iQ to perform echocardiograms on 75 divers to study the indicators of venous gas emboli associated with decompression sickness [65]. The Eftedal–Brubakk VGE grade consistency kappa value between the Butterfly iQ and the standard device Vivid q (GE Healthcare Technologies, Inc., Chicago, IL, USA) was 0.52 ± 0.06 (*n* = 141), indicating a moderate level of agreement between the two. This moderate consistency is partly attributed to the challenges of collecting data in the field using a larger probe. Vaidya et al. conducted a study on heart failure patients (POCUS-JVD study) at the University of Kentucky from July 2021 to March 2022 [66]. Ultrasound examinations of the internal jugular vein (IJV) were performed on 176 patients using the Butterfly iQ+ ultrasound system, which could be completed quickly within five minutes. This method assesses right atrial pressure (RAP) by measuring the respiratory variation diameter of the IJV (IJV-RVD) and serves as a reliable indicator. Burleson et al. introduced a study in which five emergency physicians trained in point-of-care ultrasound used the Butterfly iQ device for routine clinical care in an emergency department in rural East Africa over a 10-week period [67]. The quality of ultrasound images, particularly in abdominal and musculoskeletal imaging, was very good, although it did not fully meet the standards of cart-based systems overall. The resolution and frame rate for cardiac imaging were significantly lower, performing less effectively than other functionalities, but the quality still met clinical needs. The possible reason leading to a decrease in image quality for some echocardiograms may be the size of the Butterfly iQ probe, which is larger than that of phased-array probes, occasionally posing challenges during ultrasound-guided peripheral venous access and intercostal scans. Overall, in situations where funding, space, and reliable power sources may be limited, the advantages of the Butterfly iQ compared to cart-based equipment become more pronounced. Maganti et al. utilized the Butterfly IQ3 to carry out bedside pulmonary ultrasound evaluations, which encompassed cardiovascular and pulmonary assessments, on 208 hospitalized adult patients experiencing unexplained respiratory distress [68]. It is reported that compared to standard treatment, the use of POCUS led to a decrease of 246 hospital bed days, translating to a direct cost savings of $751,537. The incremental cost-effectiveness ratio was determined to be $3055 for each hospital bed day saved.

#### 3.2.2. Lung Ultrasound Imaging

Russell et al. evaluated the diagnostic performance of pre-hospital caregivers in detecting acute heart failure (AHF) in patients with dyspnea, comparing approaches with and without lung ultrasound (LUS) performed using the Butterfly iQ portable ultrasound system with the lung preset of 15 cm depth [69]. Incorporating LUS resulted in a 39% increase in the frequency of treatment initiation for patients with AHF. Their results demonstrated that the addition of LUS substantially enhanced both the sensitivity and overall accuracy of AHF diagnosis in the pre-hospital setting. Tung et al. [70] used Butterfly iQ for LUS examinations in 51 patients with COVID-19. The experimental results demonstrated that LUS had diagnostic value comparable to chest CT in detecting lung abnormalities in COVID-19 patients, with no cases of missed diagnoses reported. Bennett et al. [71] conducted a comparative assessment of lung involvement in 34 hospitalized COVID-19 pneumonia patients using the Butterfly iQ and a standard high-end ultrasound scanner Venue GO (GE Healthcare Technologies, Inc., Chicago, IL, USA). The results indicated that there were no significant differences in the LUS scores obtained from the high-end ultrasound scanner and the portable handheld ultrasound device. Due to its portability, ease of use, and the ability to connect to compatible Apple and Android devices via Lightning or USB-C ports, the Butterfly iQ is well-suited for use in home settings, particularly for home care. Malia et al. conducted an experiment where parents performed lung ultrasound examinations at home without real-time guidance [72]. After a 15 min practical tutorial, parents were provided with a Butterfly iQ system and a tablet, allowing them to scan their children’s lungs daily for seven consecutive days. On average, parents were able to complete some daily scans on 3.8 days during the week. Regardless of their education level, they were able to obtain interpretable images. The parents reported that the program was easy to implement and highly likely to recommend it to their friends.

#### 3.2.3. Abdominal–Pelvic Ultrasound Imaging

Alfuraih et al. conducted abdominal aortic aneurysm screening for the entire abdominal aorta using the Butterfly iQ on 114 male participants [73]. The study indicated that using the Butterfly iQ for abdominal aortic screening was feasible and reliable compared to the traditional ultrasound machine EPIQ 7 (Philips Medical Systems, Bothell, WA, USA), with the added benefit of reduced examination time. Pontones et al. investigated the feasibility and acceptability of self-guided mobile ultrasound using the Butterfly iQ among pregnant women [74]. In the study, the obstetric preset frequency for the Butterfly iQ was set at 1.7/3.4 MHz, with a dynamic range of 32–48 dB. The results showed that the device provided adequate image quality for assessing amniotic fluid and fetal heart rate, and the majority of pregnant women reported a good level of acceptance, particularly when remote support from professionals was available. Bui et al. conducted bedside ultrasound training at the Georgetown Public Hospital in Guyana, a low-to-middle-income country, successfully training 20 resident physicians in urology to use the Butterfly iQ [75]. Toscano et al. carried out a study involving medical students who had not received formal ultrasound training, utilizing a low-cost portable ultrasound system for blind ultrasound scanning to diagnose pregnancy complications [76]. The trainees used the obstetric preset B-mode imaging of the Butterfly iQ to perform 194 blind ultrasound examinations on 168 different pregnant women (248 fetuses). The study indicated that this approach demonstrated excellent sensitivity and specificity for detecting high-risk pregnancy complications, comparable to the results obtained from diagnostic ultrasound examinations conducted by trained ultrasound technicians using standard ultrasound machines. Wright et al. [77] examined the reliability of the Butterfly iQ in measuring prostate and bladder volumes compared to the piezoelectric handheld ultrasound device Clarius C3 (Clarius Mobile Health, Vancouver, BC, Canada). The study revealed that the Butterfly iQ provided more reliable and accurate measurements of prostate gland volume and urinary output than the Clarius C3. Additionally, the images produced by the Butterfly iQ were subjectively clearer when displaying deeper structures, such as the prostate. The research by Gibbons et al. indicated that the diagnostic accuracy of the Butterfly iQ for examinations of the heart, lungs, biliary tract, kidneys, or abdominal aorta is comparable to that of traditional cart-based ultrasound devices such as the GE Venue Go and GE Logiq E (GE Healthcare Technologies, Inc., Chicago, IL, USA) [78]. In the study by Araujo et al., it was found that under the operation of imaging specialists, the Butterfly iQ excelled in distinguishing between intrauterine devices positioned correctly within the uterine cavity and those that were mispositioned [79]. It also performed well in differentiating between various subtypes of mispositioned intrauterine devices. Compared to the gold standard of transvaginal ultrasound, the agreement rate between these two methods was 95.7%, with a kappa value of 0.87.

#### 3.2.4. Neuromusculoskeletal Ultrasound Imaging

In a study involving adolescent long-distance runners, Dejong et al. [80] utilized the Butterfly iQ+ at 10 MHz to evaluate changes in lower limb tendon thickness, tissue echogenicity, and muscle pennation angle in adolescent runners participating in a 6-month long-distance training program. B-mode ultrasound imaging was performed on the lower limb muscle-tendon structures, including the medial gastrocnemius, tibialis anterior, flexor digitorum brevis, and abductor hallucis, as well as the short and long axes of the Achilles tendon and patellar tendon, to assess the morphological changes induced by training. Elliott et al. investigated the inter-device reliability of the handheld Butterfly iQ+ compared to the existing ultrasound instrument SonoSite M-Turbo (FUJIFILM Sonosite, Inc., Bothell, WA, USA) in measuring muscle thickness during sustained contraction of the lumbar multifidus in patients undergoing rehabilitation for lumbar spine disorders [81]. Examinations conducted on 42 participants demonstrated that the Butterfly iQ+ exhibited excellent reliability in measuring lumbar muscle thickness. Additionally, measurements taken by new users across different devices showed good intraclass correlation. The study findings indicated that the Butterfly iQ+ is effective for imaging and measuring lumbar discs in a clinical setting. Corte et al. carried out a study to evaluate the accuracy and performance of the handheld ultrasound device Butterfly iQ in comparison to the cart-based ultrasound device Samsung HS40 (Samsung Medison Co., Ltd., Seoul, Republic of Korea) for assessing joint and periarticular lesions in patients with inflammatory arthritis [82]. In a study involving measurements of 186 joints and corresponding tendons/insertions in 32 patients, the Butterfly iQ demonstrated a total agreement of 97% with the traditional cart-based device in assessing intra-articular effusion using B-mode imaging, with a kappa coefficient of 0.90. However, the power Doppler mode of the Butterfly iQ showed limitations in detecting blood flow around the joints and adjacent areas. The study by Burleson et al. also indicated that the handheld Butterfly iQ provides high-quality ultrasound imaging for musculoskeletal evaluations [67]. Dillon et al. also indicated that both novice and experienced physical therapists are able to reliably use the handheld Butterfly iQ+ to measure the thickness of the lumbar multifidus and transversus abdominis [83]. Wheat et al. investigated the accuracy of handheld ultrasound devices in diagnosing nerve involvement in patients with leprosy [84]. Ultrasound examinations of the bilateral median nerve, ulnar nerve, C5 nerve root, and greater auricular nerve were conducted on eight leprosy patients using the standard ultrasound instrument LOGIQ e (GE Healthcare Technologies, Inc., Chicago, IL, USA) at a frequency of 15 MHz and the Butterfly iQ at a frequency of 5 MHz. The cross-sectional areas of the nerves measured by both devices were compared. The study revealed a strong correlation between the measurements from the two devices, indicating that the handheld ultrasound device can effectively identify nerve thickening in patients with leprosy. Given its portability and low cost, such devices may facilitate the diagnosis of leprosy in areas with limited medical resources.

#### 3.2.5. Image Quality and Thermal Limitations

The Butterfly iQ series represents the most successful commercial example of CMUT-on-CMOS technology. Although the Butterfly iQ series has achieved a successful demonstration in the field of general medical imaging, there are still some limitations. One notable aspect is the image quality, which is influenced by the output acoustic pressure characteristics of CMUT transducers, affecting the achievable SNR in pulse-echo imaging. Le et al. compared four commonly used handheld ultrasound devices in POCUS applications, and in terms of overall image quality satisfaction, the Butterfly iQ+ ranked lower [85]. In the cross-sectional study conducted by Perez et al., which compared POCUS across six handheld ultrasound devices, the Butterfly iQ+ also received lower scores in image quality [60]. These observations highlight that, although CMUTs have advantages in integration with CMOS circuits that help optimize signal paths; the output acoustic pressure properties can influence the achievable SNR in medical imaging applications.

Another issue is the thermal limitation. Although CMUTs are silicon-based electrostatic devices whose self-heating and heat dissipation performance are better than those of traditional piezoelectric materials, they generate more heat under high duty cycle driving or continuous operation modes, such as color Doppler imaging. In addition, the CMOS circuitry also produces significant heating. Therefore, this brings about trade-offs in thermal management and signal processing within the highly integrated systems. Burleson et al. mentioned that the resolution and frame rate of Butterfly iQ in echocardiography significantly decrease when using color Doppler [67]. There is also the issue of periodic device overheating, which leads to the inability to continue scanning until the device cools down. The Butterfly iQ series user manual indicates that the automatic cooling function activates when the probe temperature reaches 43 °C, and scanning resumes only after the estimated probe temperature decreases to 38.5 °C [86]. In high-power preset modes, the probe supports continuous scanning for approximately 10 to 25 min at an ambient temperature of approximately 25 °C.

## 4. Applications of CMUTs in Photoacoustic Imaging

### 4.1. Principle of Photoacoustic Imaging

**Figure 5:**
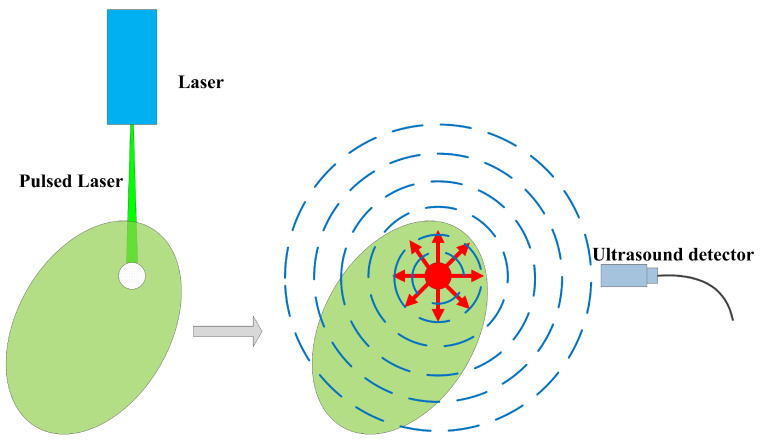
Principle of photoacoustic imaging.

Photoacoustic imaging is a rapidly developing and highly promising non-invasive biomedical imaging modality based on the principle of the photoacoustic effect. It allows for imaging the internal structural morphology of biological tissues, combining the advantages of high optical image contrast and deep ultrasound penetration. The principle of photoacoustic imaging is depicted in Figure 5. When absorbers with different optical absorption coefficients in biological tissues are irradiated by a laser, they absorb energy and convert it into heat. This heating causes thermal expansion and contraction, resulting in vibrations that emit ultrasound waves, a phenomenon known as the photoacoustic effect. The intensity and frequency of the ultrasound waves are closely related to the physical properties, optical absorption characteristics, and elastic features of the irradiated objects. By using ultrasound transducers to collect photoacoustic signals, it is possible to reconstruct 2D or 3D images of biological tissues based on relative optical absorption coefficients using imaging algorithms.

CMUTs show significant advantages in photoacoustic imaging, particularly in photoacoustic computed tomography (PACT) and photoacoustic microscopy (PAM), due to their high bandwidth, flexible design for device arrays, and wider acceptance angles [62,87]. In addition to the aforementioned advantages, CMUTs can support multi-frequency operation and transparency, which enhances their potential applications in biomedical photoacoustic imaging.

### 4.2. Traditional CMUTs in PACT

PACT typically employs one or more unfocused ultrasound transducer arrays to receive photoacoustic signals from multiple angles within the tissue. These signals are then processed using computed tomography reconstruction algorithms to generate three-dimensional images of the light absorption distribution [88]. The reconstruction goal of PACT is to recover the light absorption distribution throughout the entire imaging area, allowing it to provide a larger field of view and greater penetration depth [89].

Wygant et al. and Vaithilingam demonstrated PACT experiments using a 2D CMUT array with 16 × 16 elements in 2005 and 2006, respectively [90,91]. They embedded polyethylene tubes filled with ink inside tissue-mimicking material phantoms as the light absorbers. Subsequently, Vaithilingam et al. [92] utilized a 2D CMUT array with a center frequency of 3.48 MHz to achieve 3D photoacoustic imaging of two types of phantoms, which included alternating black and transparent fishing lines with diameters of 180 μm and 150 μm, as well as polyethylene tubes embedded in chicken breast tissue. Under a 64 × 64 aperture, the axial resolution for imaging a catheter at a distance of 1.8 cm was 300 μm, while the lateral resolution was 648 μm. Ma et al. [93] conducted a 3D photoacoustic tomography experiment using a 2D CMUT array with a central frequency of 5.5 MHz, consisting of 16 × 16 elements. They simulated a moving 64 × 64 elements array to image three horsehairs, each 150 µm in diameter, embedded at different depths within a background medium consisting of Intralipid combined with agar. 3D volumetric images and 2D maximum intensity projection images were reconstructed with an axial resolution of 680 μm and the lateral resolution of 1200 μm when the target depth was 5 cm. To enhance the imaging depth of photoacoustic imaging, Kothapalli et al. integrated pre-amplifier circuits with a 2D CMUT array using flip-chip bonding methods [94]. Using a 16 × 16 element array with a center frequency of 5.5 MHz, they performed photoacoustic imaging of three horsehair strands, each with a diameter of 100 μm, placed at depths of 2.2 cm, 3.1 cm, 4.1 cm, and 5.3 cm within a chicken breast phantom. At a target depth of 5 cm, the imaging achieved an SNR of approximately 35 dB, with an axial resolution of 500 μm and a lateral resolution of 360 μm, respectively. Cheng et al. [95] developed a minimally invasive photoacoustic signal receiver using a CMUT array, which is integrated onto a miniaturized silicon chip with a thickness of approximately 100 μm and dimensions ranging from 2.8 mm × 8 mm to 5 mm × 18 mm. The array has a center frequency of 5 MHz with a 6 dB fractional bandwidth of 116%. Vallet et al. [62] compared commercial PZT ultrasound transducers with CMUT probes manufactured by Vermon and ACULAB for photoacoustic imaging of a suture wire and bimodal phantoms. Their quantitative assessment showed that the SNR and contrast-to-noise ratio for CMUT probe imaging were improved by at least 6 dB compared to the PZT probes, and the CMUT probe also exhibited a broader acceptance angle.

To improve the field of view, resolution, and contrast of CMUT-based photoacoustic imaging, Gholampour et al. proposed a multi-CMUT approach on a flexible array that enables multi-perspective viewing via shared channels [96]. Imaging is performed by time-multiplexing the array signals through switching the DC bias voltages; the multiplexed signals are reconstructed from the CMUT and then combined coherently or incoherently. In vitro phantom experiments indicated that, compared with a single CMUT, using three CMUTs achieved about a 20% increase in generalized-contrast-to-noise ratio, a 2 dB increase in peak SNR, and a 2 times expansion of spatial coverage [97]. To further improve the SNR and frame rate of this method, Gholampour et al. subsequently proposed an encoding technique for multi-aperture photoacoustic imaging, aimed at improving SNR without significantly sacrificing frame rate [97]. Phantom imaging experiments showed that using three transducers with S-sequence encodings yields 1.5 dB improvement in SNR.

### 4.3. Multi-Frequency CMUTs in PACT

The photoacoustic signals generated from a small optical absorber exhibit a bipolar shape, featuring both positive and negative amplitudes [98,99,100]. The temporal characteristics, such as pulse duration $\tau_{a}$, and the frequency characteristics, specifically the half-power frequency range, are calculated as follows.

(4) $$\tau_{a}=\frac{2a_{s}}{c}$$

(5) $$f_{l}=\frac{0.16c}{a_{s}},\;f_{u}=\frac{0.51c}{a_{s}}$$

In these equations, $a_{s}$ is the diameter of the target sphere, *c* is the propagating velocity of the photoacoustic signal, $f_{l}$ and $f_{u}$ are the low and high frequencies for half power. It can be found that a small sphere produces a high-frequency spectrum, while a large sphere generates a low-frequency spectrum. Therefore, an ultrasonic transducer with a wide bandwidth is advantageous because it captures different frequency spectra that reflect the characteristics of absorbers of varying sizes. Compared to piezoelectric ultrasonic transducers, CMUTs not only offer a broader bandwidth but also allow for the combination of CMUT cells with different dimensions on a single probe using photolithography techniques. This capability enables functionality across multiple frequency bands.

In 2017, Chee et al. [101] first designed multi-frequency CMUTs for photoacoustic imaging. They developed surface micromachining technology to fabricate interleaved CMUT elements with two different frequencies, 1.74 MHz and 5.04 MHz, within a single array. The low-frequency element exhibited a fractional bandwidth of 134%, while the high-frequency element had a fractional bandwidth of 90%. The array was immersed in vegetable oil for photoacoustic imaging, where a single hair was utilized to simulate small optical absorbers resembling microvessels, and a piece of dyed dental floss was used to represent a larger target with a diffused optical reporter distribution. Experimental results showed that the high-frequency elements provided better resolution, making it suitable for imaging vascular structures, while the low-frequency element offered a higher SNR, making it appropriate for imaging larger, slowly varying contrast agents.

In 2017, Zhang et al. were the first to utilize a dual-frequency CMUT array for photoacoustic imaging for an in vivo study [102]. They developed surface micromachining technology to design dual-frequency CMUT elements operating at 4 MHz and 10 MHz in one array. PACT was performed on zebrafish using both the dual-frequency CMUT array and commercial piezoelectric ultrasonic transducers with similar frequencies (V382 and V311, Olympus NDT Inc., Waltham, MA, USA). The experimental results demonstrated that both low-frequency and high-frequency CMUTs in the array exhibited superior bandwidth and larger half-acceptance angle compared to the corresponding piezoelectric transducers. The low-frequency CMUT elements enabled high-quality imaging of the zebrafish body as well as major organs and structures, including the eyes, mouth, swim bladder, and fins. In contrast, the high-frequency CMUT elements provided excellent visualization of fine organ details, such as the swim bladder structures, stripe patterns, and mesenchymal tissues. This work demonstrated that combining photoacoustic signals from both the high-frequency and low-frequency ranges is crucial for accurately characterizing the physiological structures of zebrafish.

In 2018, Pun et al. integrated five different CMUT elements into one array, with frequencies of 2.9 MHz, 3.7 MHz, 5.3 MHz, 7.0 MHz, and 9.3 MHz [103]. It covered a bandwidth range from 1.8 to 10.6 MHz. The array was then used for PACT imaging of the brain in vivo in a nude mouse. The imaging results demonstrated that low-frequency CMUTs achieved higher SNR, clearly revealing the contours and shapes of brain tissues and large blood vessels, but lacking detailed structural information. In contrast, high-frequency CMUTs exhibited lower SNR, with slightly blurred brain tissue contours, yet were able to clearly display the cerebral superior veins. The study indicates that integrating multiple frequency CMUTs on a single probe can simplify the manufacturing challenges of broadband transducers used for receiving photoacoustic signals in biological tissue (PACT).

In 2023, Ghavami et al. fabricated a dual-frequency CMUT array with center frequencies of 4.2 MHz and 9.3 MHz using optically transparent materials based on the adhesive wafer-bonding method [104]. Compared to only using low and high frequencies, the dual-frequency operating mode with center frequencies of 4.2 MHz and 9.3 MHz increased the fractional bandwidth by 60% and 79%, respectively. Photoacoustic imaging results also indicated that the low-frequency components provided a higher SNR at greater depths, while the high-frequency components offered finer spatial resolution.

The above studies indicate that using CMUT-based multi-frequency ultrasound detectors is a feasible solution for photoacoustic imaging of biological tissues with structures of varying scales. First, CMUTs have a wide fractional bandwidth. Second, CMUTs are fabricated using photolithography techniques, allowing for the design of CMUT cells with various scales on the same layout. This enables the combination of multiple frequencies within the same fabrication process. With appropriate optimized design, the bandwidths of the CMUT elements at different frequencies can overlap, thereby broadening the equivalent bandwidth of the ultrasound detector. However, due to the varying collapse voltages of CMUTs with different dimensions, the DC bias voltages required for the operating points of different CMUTs are not the same. Therefore, this approach may introduce the need for multiple DC bias voltage controls, thereby increasing the complexity of the circuitry.

### 4.4. Transparent CMUTs in PACT

CMUTs offer the benefit of transparency in ultrasonic transducers, which is advantageous for photoacoustic imaging, notably in photoacoustic microscopy applications. Traditional photoacoustic microscopy systems typically use opaque ultrasonic transducers to detect photoacoustic signals. To avoid obstructing the optical path, they are commonly configured with central-aperture through-transmission, reflection [105], and off-axis arrangements [106]. These approaches introduce several drawbacks, including limited imaging depth, bulky systems with increased complexity, reduced spatial resolution, and low SNR of the detected photoacoustic signals [107]. CMUTs are fabricated using MEMS processes, employing optically transparent materials in the device structure, thereby realizing optically transparent ultrasonic transducers and enabling multi-frequency operation [104].

Cheng et al. [95] fabricated CMUTs for minimally invasive photoacoustic imaging using a silicon substrate with a thickness of only 100 μm. The silicon substrate and the dielectric membrane are relatively transparent to near-infrared light, which helps to reduce or eliminate shadowing issues related to piezoelectric ultrasonic transducers. Zhang et al. [108] utilized glass as the substrate and ITO as the electrode material to fabricate CMUTs. They employed the anodic bonding process to fabricate CMUTs with the vibrating membrane of 2 μm silicon. Compared to CMUTs that use Cr/Au as the bottom electrode, these CMUTs showed an approximately 300% increase in light transmittance in the visible to near-infrared wavelength range (400 nm to 1000 nm). Zhang et al. fabricated CMUTs for backward-mode photoacoustic imaging using borosilicate glass as the substrate, ITO as the electrode material, and a vibrating membrane made of 1.5 μm silicon, employing an anodic bonding process [109]. These CMUTs exhibited the transparency of 30–60% in the wavelength of 700 nm–900 nm, the center frequency of 1.4 MHz, and a fractional bandwidth of 105%. Photoacoustic imaging was conducted on phantoms consisting of a pencil lead immersed in oil and a polyethylene tube filled with indocyanine green (ICG) solution.

Li et al. proposed transparent CMUTs using a glass substrate and ITO electrodes, with silicon nitride as the vibrating membrane and optically transparent photo-BCB (Cyclotene 4022-25) as the adhesive and structural material, fabricated through adhesive wafer-bonding technology [110]. These CMUTs exhibited the center frequency of 2 MHz with a fractional bandwidth of 52.3%, and a maximum transparency of 82% within the visible light range. Ilkhechi et al. used a similar method to fabricate arrays containing 64 and 128 elements [111]. The average transparency of the devices in the visible light wavelength range is 70%, while it reaches up to 90% in the near-infrared range (830 nm). The photoacoustic imaging SNR for the 128-element array is 40 dB, with the lateral resolution of 234 μm and the axial resolution of 220 μm, respectively. In another study, Kashani et al. used fused-silica wafers as the substrate while maintaining similar fabrication processes as previously described. They developed a 128-element transparent ultrasonic transducer array with a center frequency of 9 MHz and a fractional bandwidth of 150% [35]. The transparency of CMUTs reached up to 90% within the visible light wavelength range. This study was reported as the first use of a transparent CMUT array for real-time combined optical and ultrasound imaging.

Ghavami et al. utilized BCB-based adhesive wafer-bonding technology to fabricate dual-frequency transparent CMUTs with interlaced low-frequency 4.2 MHz and high-frequency 9.3 MHz channels to reduce the grating lobes in the point spread function during photoacoustic imaging [104]. In addition, Ghavami et al. were the first to present the fabrication and characterization of a 64-channel flexible transparent CMUT array for use in through-illumination mode PACT [112]. CMUTs were fabricated using adhesive bonding with a polydimethylsiloxane (PDMS) backfill method. The curvature radius of this array is less than 5 mm, with a maximum transparency of 67% in the visible light range. In the PACT imaging experiment of a 100 μm metal wire target, it was found that the CMUTs exhibited a lateral resolution of 293 μm, an axial resolution of 382 μm, and an SNR of 46 dB. Additionally, PACT imaging was also performed on an ex vivo chicken breast using a through-illumination method. Table 5 summarizes the features of transparent CMUTs that can be used in photoacoustic imaging.

Apart from the aforementioned research, SU-8-based adhesive wafer bonding has been investigated to achieve transparent CMUTs [30]. The resonance frequency of CMUTs in air is 62 kHz, making them suitable for air-coupled applications. Adhesive wafer bonding with transparent polymers is a mainstream method for realizing transparent CMUTs, because the transparent photosensitive polymer materials can be used not only for wafer bonding but also for defining the cavities in the CMUT structure. Additionally, they offer good tolerance to wafer surface defects and contamination during bonding, which improves fabrication yield [29].

### 4.5. Photoacoustic and Ultrasonic Dual-Modal Imaging

Photoacoustic and ultrasound dual-modal imaging is an emerging biomedical imaging technology that combines the high contrast of photoacoustic imaging with the high-resolution and deep penetration of ultrasound imaging, enabling comprehensive information about tissue in terms of function, structure, and vascular dimensions. It operates by converting optical energy into ultrasound signals, thereby addressing the depth limitations of pure optical methods and the contrast shortcomings of conventional ultrasound in soft tissues [113].

Nikoozadeh et al. [114] used a CMUT phased-array catheter for IVUS and photoacoustic dual-modality imaging. The catheter includes a 24-element linear array and a 64-element ring array, both with a center frequency of 10 MHz. The catheter has a diameter of 3 mm. To achieve photoacoustic imaging, an optical catheter with an outer diameter of 8 mm was used to couple the laser. In the experiments, lipid emulsion imaging was performed with embedded pencil leads and with both dark-colored and transparent nylon fishing lines, and in vivo imaging of a mouse kidney was conducted. Asao et al. report the PAM-02 ultrasound and photoacoustic dual-modality breast imaging system [115]. The detector for photoacoustic signals is a 600-element CMUT array with a center frequency of 2 MHz and a fractional bandwidth of 130%. In human breast cancer examinations, PAM-02 showed improved spatial resolution and a better contrast-to-noise ratio compared with the first-generation PAM-01, which used a piezoelectric ultrasound transducer. PAM-02 can analyze vascular properties near tumors but remains challenging for detecting certain lesions. Based on this work, Shiina et al. developed PAM-03 photoacoustic imaging using hemispherical sensors for clinical breast cancer diagnosis, reducing limited-view artifacts to enhance visualization of three-dimensional vascular structures [116]. The system includes 512 CMUT elements with a diameter of 3 mm. The spatial resolution of the photoacoustic images is 0.57 mm. Matsumoto et al. developed a high-resolution photoacoustic tomography system named PAI-04, which can perform laser irradiation with inter-pulse wavelength switching, enabling the acquisition of 3D photoacoustic and volume ultrasound images [117]. The system integrates 500 CMUT elements with a center frequency of 4 MHz and a fractional bandwidth exceeding 100%. It achieves a spatial resolution of 0.27 mm and can distinguish small arteries and veins within breast tumors.

## 5. Challenges

Although CMUTs have significant advantages in terms of broad bandwidth, compatibility with CMOS fabrication processes for easy integration with AFE, and high design flexibility, there are certain limitations that restrict the further application of CMUTs in medical imaging.

### 5.1. Lower Output Acoustic Pressure

The relatively low output acoustic pressure is one of the major drawbacks of CMUTs. An early pulse-echo study of ultrasonic transducers showed that the CMUT arrays exhibited approximately 10 dB lower loop gain than the PZT arrays [118]. Recent comparative evaluations of several handheld ultrasound devices in POCUS applications have shown that the Butterfly iQ+ exhibits poorer imaging quality, primarily due to its CMUT transducer producing lower output pressure [60,85].

CMUTs operate on the principle of electrostatic actuation of a capacitive plate, and the output pressure is tightly related to the membrane’s vibration displacement. Insufficient membrane displacement is a key factor limiting the output pressure. In the transmit mode, the maximum membrane displacement mainly depends on the gap height. Enlarging the gap height can increase the maximum membrane displacement in transmit mode. However, a smaller gap height is desirable in the receive mode because it improves sensitivity. Therefore, the gap height optimization involves a trade-off in CMUT design. In addition, given a fixed gap height, the size, structure, and materials of the membrane also affect the vibration displacement. Therefore, research aimed at improving the output pressure of CMUTs mainly focuses on these factors.

#### 5.1.1. Unconventional Operating Modes

**Figure 6:**
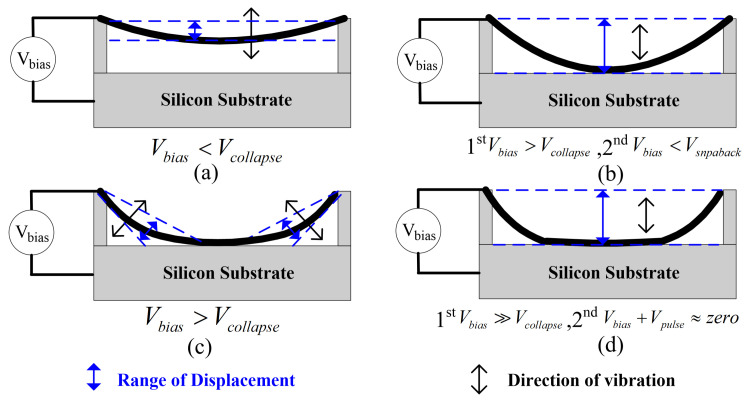
Operation modes of CMUTs: (**a**) convention mode; (**b**) collapse-snapback mode; (**c**) collapse mode; (**d**) deep-collapse mode.

For conventional CMUTs, the membrane’s steady-state deflection remains less than one-third of the initial gap height when the total applied voltage (AC+DC) is below the pull-in voltage. To circumvent this restriction, some other operating modes with higher bias voltages are developed that can improve the maximum membrane displacement in transmission. These unconventional operating modes include collapse-snapback [119], collapse [120,121,122,123], and deep-collapse [124], which can be distinguished by the applied bias voltages. Figure 6 depicts the differences between these operation modes.

In collapse-snapback mode, the membrane contacts the substrate and releases intermittently, with the bias voltage higher than the collapse voltage and below the snapback voltage. Therefore, the maximum vibrating displacement of the membrane covers the whole gap height, which enhances the output pressure, shown in Figure 6b. Bayram et al. reported that the output power improved by 83.3% for collapse-snapback CMUTs compared with conventional mode [119]. CMUTs in collapse-snapback mode deliver higher output pressure but poorer linearity than other modes, making them potentially suitable only for transmit-only applications such as therapy.

In collapse mode, the DC bias exceeds the collapse voltage, keeping the membrane collapsed and attached to the substrate throughout operation. The CMUT generates ultrasound by applying an AC voltage at the bias point, with the total biased voltage kept above the snapback voltage, as shown in Figure 6c. In collapse mode, the vibrating region is the annular portion between the center and the rim, not the whole membrane as in conventional mode, leading to a higher center frequency. Because the gap height is reduced during operation, the capacitance and electric field strength increase, resulting in higher transmission and receive performance than in conventional mode. Oralka et al. reported an experiment on a 2D high-frequency CMUT array showing that the collapse-mode CMUT had a maximum peak-to-peak pressure 59.5% higher than in the conventional operating mode [121]. In an experiment on a 1D low-frequency CMUT array, Huang et al. found that the collapse-mode CMUT exhibited a maximum output pressure 94.0% higher than a CMUT operating in conventional mode at the same bias voltage [122]. In another comparison experiment using a 1D phased array, Park et al. indicated that the output pressure in collapse mode was 107.9% higher than in conventional mode at the same frequency of 10 MHz [123]. Although CMUTs in collapse mode can enhance output pressure, the center frequency also shifts upward, which can be adjusted by the DC bias voltage. In addition, the fractional bandwidth is also affected.

In deep-collapse mode, the DC bias voltage far exceeds the collapse voltage. Firstly, a large positive DC bias deeply collapses the membrane, and then a large negative pulse is applied to the top electrode potential to zero to release it, as shown in Figure 6d. To achieve a higher pressure, the collapsed membrane is excited by a high-amplitude pulse as large as possible. Although there was no direct comparison of output pressure between CMUTs operated in deep-collapse mode and those in conventional mode reported in experiments, the output pressure per unit voltage in deep-collapse mode (48 kPa/V) [124] is higher than in collapse mode and collapse-snapback mode (10–27 kPa/V) [119,121,122,123].

Beyond the above modes, Wong et al. introduced a CMUT implementation method for HIFU applications [125]. A peak-to-peak output pressure of 1.4 MPa was obtained by applying a DC bias voltage of 172 V (70% of the pull-in voltage) and an AC voltage exceeding 100% of the pull-in voltage to the 2.5 MHz CMUTs. In continuous-wave mode, when the CMUTs were driven at 2.5 MHz with a DC voltage of 196 V (80% of the pull-in voltage) and an AC voltage of 250 Vpp for over 1 h, the peak-to-peak output pressure of 1.7 MPa was measured.

#### 5.1.2. Unconventional Membrane

Because the pressure is generated by the vibrating membrane of CMUTs, the displacement of the membrane is a key factor for output pressure. One possible approach involves designing a modified membrane with a mass at its center, which changes the membrane’s geometry in the vertical direction to enable piston-like motion [126,127,128]. In comparison to conventional CMUTs, this nonuniform membrane with a central mass demonstrates greater average displacement, leading to higher output pressure. Huang et al. conducted a comparison between a CMUT featuring a square uniform membrane and one that incorporates a square mass at the center of the membrane [126]. It showed that the output pressure was increased by 82.5% and the reception sensitivity was improved by 95.0%, respectively. Yoon et al. performed a direct comparison of a single cell between a conventional CMUT and one with a gold center mass in oil. The results showed that the maximum output pressure of the latter was improved by 23.4% [128].

In conventional CMUTs with uniform membranes, the vibrating membrane exhibits complex modal behaviors, including the first-order mode and higher-order modes. The center-mass structure significantly lowers the resonant frequency of the first-order mode while shifting the higher-order modes to higher frequency ranges, thereby making the piston-like mode the dominant vibration shape. The center mass increases the local inertia of the membrane, which reduces the vibration amplitude near the edges and promotes more uniform overall motion. This results in a larger average volume displacement and, consequently, higher output pressure. The piston-like motion produces a more uniform velocity distribution across the membrane, bringing the radiation impedance closer to that of an ideal piston radiator. As a result, acoustic impedance mismatch is reduced, and the bandwidth may also be improved. Refs. [126,127] have reported enhanced bandwidth in center-mass CMUT designs. Furthermore, the piston-like vibration concentrates the acoustic energy of each CMUT cell more effectively in the vertical radiation direction rather than in lateral bending or shear waves. This reduces the mechanical energy transmitted through the substrate or filling materials, which helps to lower crosstalk between array elements.

Forming trenches in the membrane is also an effective approach to improve output pressure. The trenches reduce the effective thickness of the membrane, leading to a decrease in its effective flexural rigidity. Consequently, the regions with trenches will experience greater deflection, which allows for a more uniform overall displacement of the membrane by adjusting the position and number of trenches. Zhou et al. utilized finite element method (FEM) simulations, which indicated an increase of 122.2% in output pressure capability for the 4-trench CMUT design [129]. The trench structure modifies the stiffness distribution of the membrane, preventing purely bending deformation under force. This allows the membrane to move more freely as a whole, approximating piston-like motion. As a result, the average volume displacement of the membrane increases, leading to higher output pressure. This structure enlarges the effective radiating area and improves acoustic impedance matching with the medium and provides better bandwidth. Furthermore, the concentrated vertical vibration reduces lateral energy coupling, thereby minimizing crosstalk between adjacent array elements.

Lee et al. reported a CMUT with a substrate-embedded spring structure in which the embedded silicon springs support a thick silicon piston plate [130]. Under electrostatic forces, this plate vibrates like a piston, enhancing the membrane’s average displacement. Although this CMUT structure was not directly compared with a conventional CMUT in output pressure, its maximum output pressure efficiency exceeds that of the HD3203 PZT, the PZ21 PZT, and a commercial 1D PZT phased-array probe.

Elshenety et al. reported FEM studies on increasing CMUT output pressure with various approaches [131]. They optimized the membrane radius, thickness, and gap height; compared the use of square waves and sine waves in transmission; explored dielectric layers with higher permittivity; and investigated membranes with trenches or masses.

#### 5.1.3. Unconventional Electrodes

Besides the methods of modifying the membrane structure, there is another approach to alter the structure of the electrodes, which involves changing the single top electrode into a dual-top-electrode configuration. This structure separates the top electrode into two side electrodes that are interconnected and connected to a power source for transmission, reception, and membrane shaping. Additionally, there is a center electrode connected to another power source, primarily for receiving operations [132,133,134]. By utilizing the effect known as leveraged bending [135], the stable range of the membrane can be enhanced through the activation of the side electrodes. The transmission swing range nearly encompasses the entire gap without causing the membrane to collapse. Additionally, the membrane can be precisely adjusted by the side electrodes, allowing for a reduced distance between the center top electrode and the bottom electrode during the receiving operation mode. Guldiken et al. reported that in the experiments, the dual-top-electrode CMUTs demonstrated an improvement of 6.8 dB (or 118%) in maximum output pressure when side electrodes were used alongside the conventional center electrode [132]. Guldiken et al. later demonstrated that by employing dynamic membrane shaping, the CMUTs achieved a 7.4 dB (or 142.4%) enhancement in maximum output pressure, along with a 16.4 dB improvement in overall performance compared to conventional CMUTs [133]. In addition, the output pressure can be further increased by 2 dB by introducing a nonuniform membrane compared with a dual-electrode CMUT with a uniform membrane [134].

In the dual-top electrodes design, because the gap height beneath the center electrode is smaller, the DC bias voltage required for the center electrode to operate in receive mode is significantly reduced. Another advantage is that the DC bias voltages of the center electrode and the side electrodes can be controlled independently, allowing the transmit and receive modes to be optimized separately. This enables the membrane to have a larger effective gap during the transmit mode and a smaller effective gap in the receive mode. Consequently, both the output pressure and the receive sensitivity are improved simultaneously.

An alternating electrode structure was implemented to divide the bottom electrode into dual-bottom electrodes, rather than modifying the top electrode as previously mentioned. FEM simulations indicated that this configuration yielded improved performance compared to conventional CMUTs, resulting in a 5.7 dB (or 91.7%) increase in output pressure during transmit mode and an enhancement of 9.3% in reception sensitivity [136].

Li et al. designed a CMUT with annular electrodes that exploits electrostatic spring softening to selectively reduce the stiffness of the outer membrane region while preserving the central stiffness [137]. This nonuniform stiffness distribution enables piston-like membrane deformation and increases the output pressure. FEM analysis showed that at the same bias ratio, the annular-electrode CMUTs achieved up to 255% higher output pressure than conventional CMUTs in air-coupled scenarios. In this design, the pull-in voltage varies with the area covered by the annular electrode. Compared with other modified CMUT structures designed to enhance output pressure, its advantages lie in the simple structure and straightforward fabrication process.

#### 5.1.4. Unconventional Cavity

The gap provides the space for membrane vibration and affects the electric field strength between the electrodes. The output pressure of CMUTs can also be increased by altering the gap structure. Li et al. introduced a CMUT featuring a T-shaped gap structure with a smaller edge and a higher central region [138]. By leveraging electrostatic stiffness softening, the outer region of the vibrating membrane experiences a larger stiffness reduction, resulting in piston-like deformation of the membrane. FEM analysis indicated that, compared to conventional CMUTs, the T-shaped CMUTs achieved 56.1% improvement of output pressure at the same bias ratio and 371.0% improvement at the same bias voltage in air.

Subsequently, Jia et al. extended the T-shaped configuration to CMUTs with stage cavities, introducing the “2-stage-Optimal” and “3-stage” designs [139]. FEM analysis indicated that the “3-stage” CMUT exhibited slightly better electromechanical coupling and transmission, and reception performance than the 2-stage-Optimal design, but has a lower input impedance and involves a more complex fabrication process, increasing cost.

#### 5.1.5. Unconventional Membrane–Electrodes Combination

Yuan et al. studied a CMUT with a combination of annular electrodes and membrane grooves. The ring electrodes adjust the membrane stiffness, and the grooves relieve edge stress to enhance membrane displacement [140]. The FEM analysis revealed that the membrane displacement, transmitting vibration amplitude, and receiving sensitivity increased. The fabricated device experiment showed that the output pressure of the CMUTs was 37% higher than that of conventional CMUTs. Kim et al. designed a clamped CMUT composed of a circular base plate, an annular middle plate, and an annular top plate [141]. This configuration allows the circular base plate to vibrate piston-like, increasing the inter-electrode gap variation. Experimental tests showed that at the same conditions, the CMUT’s output pressure was 72% higher than that of a conventional CMUT. Li et al. proposed a dual-layer CMUT design consisting of a top-layer circular CMUT unit and a bottom-layer annular CMUT unit, connected by a movable support pillar to increase the total membrane deflection and reduce stiffness, enabling membranes to deform more readily under external forces [142]. FEM analysis showed that, compared with conventional CMUTs, the dual-layer CMUT exhibited a 17.7% higher transmit sensitivity.

#### 5.1.6. Unconventional Mode-Membrane Combination

Yu et al. proposed a method to form embossed patterns on the annular vibrating membrane to further increase the output pressure of collapse-mode CMUTs [143]. The FEM analysis showed that, compared with a uniform-membrane CMUT cell in collapse mode, the nickel-embossed pattern of 1 μm × 2 μm located at the optimum position achieves an 88.1% increase in maximum output pressure. Yu et al. later reported full top electrode CMUTs fabricated by surface micromachining, featuring nickel embossed patterns of 3 μm × 2 μm [144]. In both collapse-mode operations, the embossed-pattern CMUT achieves a 27.1% increase in output pressure compared to a uniform-membrane CMUT cell. The experimental result was lower than the FEM analysis, mainly due to process limitations, particularly the relatively wide pattern width.

#### 5.1.7. Multiphysics Coupling Mechanism Analysis

CMUTs convert electrical energy into acoustic energy output through the vibration of a membrane. This process primarily involves the multiphysics coupling among the electric field, mechanical field, and fluid field. This subsection analyzes the methods for enhancing the output pressure of CMUTs from a multiphysics perspective.

For methods related to the unconventional mode, the output pressure is primarily improved by controlling the bias and drive voltages. In collapse-snapback and deep-collapse operating modes, CMUTs experience membrane collapse due to sudden bias voltage changes, converting electrical energy into elastic potential energy stored in the large-deformation structure. When the voltage is suddenly removed or reduced below the critical threshold, the membrane rapidly snaps back, generating high-speed mechanical motion. The mechanical energy drives the fluid field through volumetric acceleration, achieving high output pressure. In collapse-mode CMUTs, the gap between the membrane and bottom electrode decreases significantly, resulting in a sharp increase in capacitance. From the multiphysics perspective, the electrostatic force under the same driving voltage in the electric field is greatly enhanced, improving the energy conversion efficiency from the electric field to the mechanical field. This generates larger volumetric acceleration during membrane vibration, therefore achieving higher output pressure at the same driving voltage.

In the unconventional membrane methods, the membrane structures are modified to achieve piston-like vibration, thereby enhancing the output pressure. With the center-mass-structured membrane, the motion during actuation tends to be dominated by piston-like vibration. The electrostatic force in the electric field is more uniformly converted into membrane displacement. The large volumetric displacements in the mechanical field directly drive the fluid field to generate higher pressures. In trench-structured CMUTs, the change in membrane stiffness enables the membrane to approach piston-like vibration. This allows the electrostatic force applied by the electric field to drive the membrane to produce larger deflection, which in turn leads to higher pressure in the fluid field through mechanical-fluid coupling. In substrate-embedded springs CMUTs, spring posts connected to the substrate are added beneath the membrane, enabling the membrane to achieve piston-like motion under electrostatic force. The electrostatic force from the electric field drives the membrane to produce larger deflection, ultimately achieving higher output pressure.

Unconventional electrodes, CMUTs, also achieve piston-like vibration of the membrane by modifying the electrode structure. In dual-top electrode CMUTs, the leveraged bending effect enhances the motion of the CMUT membrane, significantly increasing the effective vertical displacement and overall volume displacement of the membrane. The electrostatic force applied by the electric field is efficiently converted into a large deflection in the mechanical field, enhancing the volume displacement and thereby driving the fluid field to form higher acoustic pressure. As for the annular-electrode CMUTs, the annular electrode effectively enables the membrane to produce piston-like deformation, thereby increasing the average displacement and output pressure. That is, the electrostatic force applied by the electric field is more efficiently converted into volume displacement in the mechanical field through local softening, driving the fluid field to generate higher pressure.

In unconventional cavity CMUTs, the stepped cavity increases the electric field strength at the membrane periphery. It utilizes the electrostatic stiffness-softening effect to modulate the membrane stiffness, enabling the entire membrane to produce piston-like deformation and thereby improving the volume displacement; that is, the electrostatic force applied by the electric field is more efficiently converted into volume displacement in the mechanical field through the electrostatic stiffness-softening effect, driving the fluid field to form higher pressure.

CMUTs with annular electrode and membrane groove configurations utilize the electrostatic stiffness-softening effect of the annular electrode to modulate the membrane stiffness, while the membrane groove releases the stress at the membrane edge. This enables the membrane to produce piston-like vibration, thereby improving the volume displacement and output pressure. Indirectly clamped CMUTs achieve piston-like deformation of the circular membrane under electrostatic force through a separated suspended circular membrane and electrode structure. Dual-layer CMUTs increase the total deflection and reduce the stiffness by stacking two CMUT designs, making the membrane more prone to deformation under external forces and thus enhancing the output pressure. From the multiphysics perspective, these three methods enable the electrostatic force applied by the electric field to be converted more efficiently into volume displacement in the mechanical field, driving the fluid field to form higher pressure.

The design of collapse-mode CMUTs with an embossed pattern enhances the output pressure by increasing the displacement of the vibrating membrane in collapse-mode CMUTs. From the multiphysics perspective, under the same driving voltage, the electrostatic force in the electric field is enhanced. In the mechanical field, the optimized piston-like deformation generates greater volume displacement, enabling the electrostatic force applied by the electric field to be converted more efficiently into this enhanced mechanical response, thereby driving the fluid field to form higher pressure.

In summary, the majority of CMUT research approaches primarily improve the output pressure. by optimizing the vibration mode of the membrane in the mechanical field to produce piston-like deformation. In contrast, collapse-snapback and deep-collapse mode CMUTs focus on nonlinear transient processes, improving the output pressure. by increasing the release and conversion efficiency of transient potential energy. Collapse-mode CMUTs mainly rely on increasing the electrostatic force in the electric field to enlarge the displacement amplitude, thereby enhancing the output pressure. Meanwhile, collapse-mode CMUTs with embossed patterns not only utilize the collapse contact to enhance the electrostatic force in the electric field, but also promote piston-like deformation of the membrane through the embossed pattern. This allows the electrostatic force applied by the electric field to be converted more efficiently into volume displacement of the mechanical field.

#### 5.1.8. Summary of Methods to Improve Output Pressure

In medical ultrasound imaging, the transducer’s output pressure not only determines imaging depth but also affects image SNR. Therefore, improving output pressure has become a key focus of CMUT research, as summarized in Table 6. Since ultrasound transducers act as both a transmitter and a receiver during imaging, an ideal method should improve receive sensitivity alongside output pressure to realize a balanced optimization. The performance improvement data are derived from reference publications by various research groups. To the best of the authors’ knowledge, a comprehensive quantitative comparison across various approaches to improving the output acoustic pressure of CMUTs has not yet been systematically conducted.

During the early phase of CMUT research, approaches to increase output pressure focused on the modification of operating modes, membrane shape, and electrode configuration. In particular, collapse-mode operation, a nonuniform membrane with a center of mass, and a dual-electrode CMUT design achieve simultaneous improvements in output pressure and receive sensitivity. Following earlier work, researchers have extended the study to cavity architectures, membrane–electrode combinations, and combinations of collapse-mode operation with nonuniform membranes to further enhance output pressure. Staged cavities, such as T-shaped designs and dual-layer CMUTs, have achieved simultaneous improvements both in output pressure and receive sensitivity. It should be noted that some references do not report the improvement of receive sensitivity, and some studies remain in progress. Additionally, fabrication complexity and the feasibility of new CMUT structures should be considered.

### 5.2. Dielectric Charging Effects

In electrostatically actuated MEMS devices, dielectric charging effects are a common issue because high electric fields can drive charges into insulation dielectric materials. This dielectric charging may cause capacitance-voltage hysteresis, operating point drift over time, and reduce reliability. Charge trapping in CMUTs may originate from two main factors, fabrication-related effects and the operating mode, both producing strong electric fields within the cavity region [145]. Charging effects were observed in both surface micromachining and wafer-bonding processes due to the dielectric materials incorporated in the device structure [123]. Charging effects in the dielectric between the top and bottom electrodes cause drift in center frequency and operating sensitivity, posing a key reliability challenge for commercial CMUT product design [146]. As for CMUTs working in collapse mode, this challenge is obvious because of the high electric field strength [147]. To decrease the charging effects of CMUTs, researchers have implemented optimizations across various aspects, including materials, structures, fabrication processes, and driving methods, in order to effectively reduce or mitigate charge accumulation.

#### 5.2.1. Material Optimization

Dastidar et al. investigated the use of SiC as a substitute for Si_3_N_4_ in the membrane of CMUTs to reduce charging effects [148]. Experimental results showed that CMUT cells using SiC as the driving material exhibit approximately 20% improved output performance compared to those utilizing Si_3_N_4_. Bang et al. utilized high-k dielectric material Al_2_O_3_ as the insulation layer and used the wafer-bonding technique to fabricate high dielectric constant CMUTs [149]. Compared to conventional CMUTs that use SiO_2_ as the insulation layer, the pull-in voltage of this high-k CMUT was reduced by 11.3%, therefore decreasing the electric field strength in operation and mitigating charging effects. Elshenety et al. discussed the advantages of reduced leakage current through the use of high-k dielectric materials combined with thermal annealing, aimed at minimizing charging effects. In their study, they employed tetragonal titanium dioxide as the insulation layer material [131].

#### 5.2.2. Structural Optimization

Machida et al. [150] used SiO_2_ as the dielectric material in the cavity region and designed a CMUT with spacers beneath the vibrating membrane and a partially open structure above the spacer corresponding to the top electrode. The purpose is to prevent the vibrating membrane from contacting the cavity bottom when the DC bias exceeds the pull-in voltage, thereby reducing electric field strength, mitigating charging effects, and improving the dielectric reliability of the device. Experimental results showed that when the exciting voltage exceeds the pull-in voltage, the vibrating membrane persists for more than 6 × $10^{11}$ cycles with the receiving sensitivity fluctuating by less than 1 dB. Under the same conditions, the output pressure is still three times higher than that of conventional structures. Huang et al. proposed a CMUT design called PostCMUT, in which isolation posts placed inside the cavity replace the insulating layer used to prevent short circuits [145,151]. The CMUTs were fabricated using wafer bonding. In experiments, no charging effects were observed during operation, and the transmit and receive performances were similar to those of conventional CMUTs, thereby improving the device reliability.

Mahmud et al. [152] proposed an improved CMUT structure by fabricating insulating glass spacers inside the CMUT cavities. These spacers serve as supporting posts within the cavity, mechanically supporting the top plate in collapse mode. The insulating glass spacers reduce charging in the dielectric layer by preventing direct electrode contact in collapse-mode operation, thereby limiting charge accumulation from contact and enhancing the dielectric reliability. Dew et al. proposed a CMUT design using insulated electrode posts (EP) and isolation insulating posts (IIPs) to reduce charging effects [153]. In comparisons of conventional CMUTs, EP CMUTs, and IIP CMUTs in both conventional and collapse mode, the conventional CMUTs showed evident charging after snap-down events. In contrast, CMUTs with EP or IIP structures demonstrated notable charging robustness after over 5 × $10^{5}$ collapse-snapback cycles and 18 h of high field exposure, with negligible charging. Furthermore, the EP and IIP CMUTs did not appear to shift the operating frequency and significantly degrade transmit/receive performance.

#### 5.2.3. Driver Optimization

Adjusting the bias voltage or drive signals can reduce charging effects. For example, Lemmerhirt introduced an approach that applies periodic polarity switching (at frequencies below 100 kHz) to the bias voltage. This approach exposes the CMUT to electric fields of opposite directions but for equal periods, thereby achieving a near-zero net charging effect and effectively mitigating the charging effects observed under constant DC bias conditions in CMUT devices. This method requires no modifications to the CMUT structure or materials [154].

Yamaner et al. used AC voltage excitation at the half-resonant frequency and without any DC bias because the squared voltage term contains DC components that can compensate for the externally applied DC bias [155]. Although this method lowers charging effects, CMUTs should be optimized for membrane radius, membrane thickness, and cavity height in design. Elshenety et al. further researched this method by FEM analysis and found that using AC square waves at the half-resonant frequency can achieve higher output pressure than sine waves [131].

#### 5.2.4. Pre-Charged Method

Another approach is to use a pre-charged method in CMUT fabrication or pre-treatment to inject charges into the dielectric layer, enabling the operating point to be maintained without external DC bias and reducing charging effects.

Ho et al. [156] developed a thick buried oxide layer and a partially connected donut-shaped bottom electrode to trap charges at the CMUT center, keeping them in a floating state. By applying a DC pre-charge above the pull-in voltage, charges are injected into the floating region, generating a stable intrinsic electric field in the device gap and enabling operation under an external bias. The experiments showed that the injected charges remained effective and stable for up to 19 months. Choi et al. [157] demonstrated LOCOS-based fabrication of pre-charged CMUTs, with the insulation layer consisting of Si_3_N_4_–SiO_2_ and SiO_2_. Electron injection during the pull-in/out process creates the potential difference, and the enduring surface/trap charges allow zero-bias operation. Annayev et al. designed a pre-charged CMUT specifically for implantable medical devices, incorporating a built-in charge storage capacitor with a floating electrode positioned between the top and bottom electrodes to enable long-term charge storage and eliminate the need for continuous external DC bias [158]. Before implantation, a DC bias is applied between the CMUT’s top and bottom electrodes, causing the floating electrode to contact the bottom electrode and store charge on it. After pre-charging, the charge remains leakage-free for up to 2 years, allowing operation without continuous external DC bias and avoiding charging effects.

Saccher et al. evaluated Si_3_N_4_ deposited by PECVD and Al_2_O_3_ deposited by ALD as charge storage layer materials for pre-charged collapse-mode CMUTs and investigated CMUTs with dielectric layers of varying thickness (BDiel) between the bottom electrode and the storage layer [159]. They found that Si_3_N_4_ has a higher charge-storage capacity than Al_2_O_3_, and that a thicker BDiel improves charge capture and retention.

#### 5.2.5. Summary and Comparison

Material optimization focuses on improving the dielectric layer by selecting suitable materials, refining deposition processes, and optimizing thickness to reduce trap density and suppress leakage currents. This approach improves reliability at the material level, making it suitable for long-term stable operation, and can be combined with pre-charged strategies to achieve zero bias. However, material choices and processing should be compatible with the fabrication process, and the optimization typically requires substantial experimental validation. Structure optimization approaches focus on altering the CMUT membrane or electrode structures or adding posts inside the cavity to reduce the dielectric exposure area in regions of high electric fields or to modify the electrical field distribution, thereby lowering charging effects. This method can significantly reduce charging effects and simultaneously improve output and receive sensitivity, bandwidth, and stability. However, it increases fabrication complexity and requires precise control of the designed and fabricated device dimensions to prevent significant changes in the resonant frequency. Drive optimization reduces charge injection or balances accumulated charges in CMUTs by adjusting the bias and excitation schemes. This approach does not require changes to the CMUT fabrication process or materials, but it may increase the complexity of the drive circuit and lead to higher power consumption. The pre-charged method enables zero or near-zero DC bias operation in CMUTs by actively injecting and storing charge on floating electrodes or storage layers to create an internal electric field. This approach eliminates the need for external DC bias, reducing power consumption and simplifying the drive circuit. However, careful consideration must be given to charge injection efficiency and long-term stability. Table 7 lists the comparison of these methods to reduce charging effects.

Each of the aforementioned methods has its own advantages and limitations. In practical applications, combining these approaches is expected to achieve superior overall performance, such as integrating structural optimization with pre-charged techniques to realize high reliability, zero-bias operation, and high output simultaneously.

### 5.3. High Bias Voltage Requirement

Unlike piezoelectric transducers, CMUTs typically require a high DC bias voltage of tens to hundreds of volts during operation. There are two main reasons for this. First, the amplitude of the DC bias voltage affects the electromechanical coupling coefficient and sensitivity of CMUTs. As the bias voltage increases, the electrostatic force acting on the membrane becomes larger, reducing the gap between the membrane and the substrate. This makes the capacitance change more sensitive to membrane displacement, thereby significantly improving the transmit and receive sensitivity [160]. For CMUTs operating in conventional mode, the DC bias voltage is typically set to 70–90% of the pull-in voltage to achieve high electromechanical conversion efficiency. Second, the electrostatic force in a CMUT is proportional to the square of the applied voltage. By applying a relatively large DC bias voltage and superimposing a small AC signal, the dominant AC components of the electrostatic force become linearly proportional to the AC signals, resulting in a more linear device response and significantly reduced distortion [161].

However, the requirement for high DC bias voltages in CMUTs also introduces significant challenges in medical imaging applications, particularly in scenarios demanding portability, low power consumption, or high reliability. First, it exacerbates dielectric charging, consistent with the charging effects discussed in Section 5.2. Second, maintaining a stable high-voltage DC bias requires a high-voltage DC supply, which is not readily compatible with modern low-voltage CMOS circuits (typically less than 5 V). Consequently, the drive circuit must incorporate voltage-boosting components such as charge pumps or DC–DC converters, increasing the circuit size and complexity as well as power consumption, which are particularly detrimental in portable, battery-powered, or compact medical ultrasound systems. Third, for external ultrasound probes, the high-voltage bias must be strictly isolated to prevent leakage currents or electric shocks in order to meet medical device regulatory requirements. Therefore, researchers have proposed several strategies to address this issue, which can be broadly categorized into two approaches: lowering the pull-in voltage and eliminating or reducing the need for an external bias voltage.

#### 5.3.1. Lowering the Pull-In Voltage

CMUT pull-in voltage depends on several factors such as the membrane material properties, gap height, electrode coverage area, insulating layer thickness, and its dielectric constant. Therefore, by adjusting the CMUT structural parameters and materials, the pull-in voltage can be reduced, enabling the device to reach its operating point at a lower bias voltage. One straightforward approach is to reduce the thickness of the membrane/insulation layer and to decrease the gap height. However, it should balance against several factors, including the center frequency, output pressure, bandwidth, and the membrane’s mechanical strength [162]. Additionally, fabrication feasibility should also be taken into account. Surface micromachining techniques are not suitable for producing CMUTs with tiny gap heights, so wafer-bonding technology is typically required instead [163]. With wafer-bonding processes, gap heights of less than 50 nm can be reliably achieved [27].

Merbeler et al. [164] fabricated CMUTs using a wafer-bonding process, achieving a vacuum gap height of 120 nm with piston-structured plates. This design successfully reduced the pull-in voltage to a range of 7.4 V to 25.0 V. Goel et al. [165] designed CMUTs with suspended membranes connected via spring arms to rocking stems using the PolyMUMPs surface micromachining process. The pull-in voltage was reduced to 46 V, compared to 326 V for conventional CMUTs with the same gap height. At 40 V bias voltage, the static displacement of this CMUT was 65 times greater than that of the conventional design. Goel et al. [166] further developed CMUTs incorporating spring arms and rocker stems in the membrane structure. This device achieved a pull-in voltage of 40 V and could operate effectively at a bias voltage of 26 V. Compared to conventional CMUTs, the required bias voltage was reduced by a factor of 6.5. Yu et al. [144] fabricated CMUTs with a nickel embossed pattern using surface micromachining processes. Compared to the conventional CMUTs with the same dimensions, this design reduced the pull-in voltage from 122 V to 88 V.

Li et al. designed a dual-layer CMUT using a wafer-bonding process, consisting of a top-layer circular CMUT cell and a bottom-layer annular CMUT cell. This design enables easier membrane deformation under external forces, achieving a 13.7% reduction in pull-in voltage compared to conventional CMUTs [142]. Li et al. [138] also proposed CMUTs with T-shaped cavities, where the electrode spacing in the peripheral regions is smaller than in the central regions. This design creates higher electric field strengths while adjusting the membrane stiffness, achieving a 36.2% reduction in pull-in voltage.

#### 5.3.2. Eliminating or Reducing DC Bias Voltage

This approach is similar to the pre-charged method described in Section 5.2.4 for mitigating dielectric charging effects. It involves storing charges in the insulation layer, a floating electrode, or a dedicated charge storage layer to generate an intrinsic electrostatic force, thereby eliminating or reducing the need for an external DC bias voltage. It provides a valuable technical pathway for CMUTs in low-power and miniaturized applications, such as in vivo imaging or implanted devices. Because the collapse-mode CMUTs offer higher electromechanical coupling efficiency, output pressure, and receive sensitivity compared to the conventional operation mode, some studies have applied the pre-charged method specifically to collapse-mode CMUTs. By charge trapping in dielectric layers or on floating electrodes, these devices can work in collapse mode at zero or low external bias voltage.

Ho et al. [156] introduced a pre-charged CMUT designed for collapse-mode operation. By applying a DC voltage higher than the pull-in voltage, charges are injected into the floating charge storage layer, generating a sufficiently strong intrinsic electric field. This enables the device to maintain collapse-mode operation without any external DC bias voltage. The pre-charged CMUT demonstrated stable zero-external-bias performance for over 1.5 years. However, this pre-charged collapse-mode CMUT operates at a relatively low center frequency of about 140 kHz. Recently, Saccher et al. presented a pre-charged collapse-mode CMUT that can operate without any external bias voltage, with sufficient built-in charge to maintain the collapsed state [167]. The device was fabricated using surface micromachining processes and incorporates a charge-trapping Al_2_O_3_ layer embedded within the dielectric between the top and bottom electrodes. Experimental characterization demonstrated that the acoustic performance of this pre-charged CMUT is nearly identical to that of a conventional CMUT operated with a 70 V external bias voltage. Compared to other reported CMUT designs, this device exhibits comparable transmit sensitivity while offering superior receive sensitivity. Accelerated lifetime tests further indicated that these devices have an operational lifetime exceeding 2.5 years at a body temperature of 37 °C.

To the best of the authors’ knowledge, as investigated by Saccher et al. using a 120 nmthick PECVD-deposited Si_3_N_4_ as the charge-storage layer, accelerated lifetime tests show that the charge retention time for pre-charged CMUTs can reach up to approximately 7.8 years under static non-operating conditions at body temperature (37 °C) [159]. The primary leakage mechanisms for pre-charged CMUTs include thermal detrapping, in which charges gain sufficient thermal energy to escape from shallow traps, leading to a leakage rate that increases exponentially with temperature. The isothermal environment of the human body provides continuous thermal activation energy, which may result in significant charge decay over a timescale of several years. Furthermore, the thin film thickness of pre-charged CMUTs facilitates charge leakage through the Poole-Frenkel emission mechanism; as the stored charge increases, the built-in electric field becomes stronger, accelerating the leakage [168]. Therefore, zero-bias pre-charged CMUTs are currently more suitable for laboratory prototypes or short-term applications, and further development is still needed to achieve long-term reliability suitable for implantable devices.

## 6. Discussion

CMUTs present several comparative practical advantages in medical diagnosis compared with traditional piezoelectric transducers. The article reviews CMUT applications in medical ultrasound imaging and photoacoustic imaging and highlights the three main challenges: lower output acoustic pressure, dielectric charging effects, and high bias voltage requirement. This section discusses how these three challenges manifest and impact different medical imaging application scenarios that use CMUTs. The primary strategies proposed to address these challenges are also discussed.

### 6.1. Practical Advantages of CMUTs in Medical Diagnosis

CMUTs can achieve higher frequencies and broader bandwidths, theoretically providing better axial resolution, which helps doctors observe fine vascular structures or tissue boundaries more clearly. At the same time, they also support complex array designs, enhancing diagnostic efficiency. However, the technological maturity of CMUTs for large-scale clinical applications still needs improvement. According to the current usage of the commercially available Butterfly iQ series, which uses single-probe integration technology with a lightweight and compact volume. One probe can replace multiple traditional ultrasound probes and is preset with various imaging modes, supporting imaging of the abdominal–pelvic, cardiovascular, lung, and neuromusculoskeletal systems. Its portability and cost-effectiveness make it an ideal tool for immediate diagnosis, telemedicine, and clinical research.

For doctors, it allows for efficient POCUS in emergency or resource-limited environments, reducing setup time and enabling quick assessments of cardiac, pulmonary, or vascular conditions, which aids in rapid diagnosis and makes examinations more convenient for patients. This is especially suitable for primary care, remote areas, emergency situations, and mobile healthcare. For patients, the small size of the probe means they do not need to maintain a fixed position for long periods or move to an examination room during scanning. Bedside examinations can shorten the overall visit process, which is particularly beneficial for patients with limited mobility, the elderly, or critically ill patients. In remote areas or telemedicine scenarios, patients can receive professional preliminary assessments near their homes. In addition, the batch manufacturing potential of CMUTs may reduce equipment costs, indirectly enabling more patients to benefit from ultrasound imaging examinations.

### 6.2. Relationship Between Medical Imaging and Challenges

As described in Section 3, CMUTs have been successfully applied in both specialized fields and general-purpose medical ultrasound imaging. The representative commercial CMUT device is the Butterfly iQ series, which has already entered routine clinical ultrasound diagnostics. Its low cost and portability allow use in diverse locations and crowded environments, making it especially suitable for resource-limited conditions, rural healthcare, emergency departments, battlefield medicine settings, and home care scenarios. However, in some comparative studies of POCUS devices, the Butterfly iQ series has demonstrated lower imaging quality than the handheld PZT ultrasound systems in several key parameters, including detail resolution, contrast resolution, penetration, clutter, and overall satisfaction.

For instance, Le et al. evaluated multiple handheld devices using expert POCUS assessments on standardized patients, focusing on key views such as the right upper quadrant view of the focused assessment with sonography in trauma (FAST), the transverse neck view, and the parasternal long-axis (PLAX) cardiac view [85]. In their study, Butterfly iQ+ generally received lower ratings for image quality parameters relative to certain PZT-based devices. Similarly, Perez-Sanchez et al. involved 35 multi-specialty POCUS experts acquiring and rating images from six handheld devices on standardized patients [60]. The evaluated views included the right upper quadrant, the cardiac apical four-chamber view, and superficial neck and lung views. Across these assessments, Butterfly iQ+ scored notably lower than several PZT-based devices.

One possible reason for the low imaging quality is that CMUTs are electrostatic ultrasound transducers, which generally produce lower output pressure compared to PZT transducers. This difference becomes particularly noticeable in high-frequency ranges or during deep tissue imaging scenarios. The lower transmitted acoustic pressure directly decreases SNR, penetration depth, contrast, and detail resolution.

To the best of the authors’ knowledge, CMUTs have not yet achieved large-scale commercialization or widespread clinical applications in IVUS catheter products. Currently, mainstream IVUS systems still use traditional piezoelectric ultrasound transducers. There are no publicly reported cases of FDA or CE-approved CMUT-based IVUS catheter products available on the market. The main reason may be related to the inherent characteristics of CMUT technology, particularly the lower output pressure and the requirement for a high DC bias voltage. IVUS imaging typically employs high-frequency ultrasound to achieve high resolution. However, attenuation in tissue is significant at high frequencies. If the ultrasound transducer outputs a low pressure level, both the imaging penetration and SNR will decrease. Furthermore, the high-voltage DC bias required for CMUT operation presents a challenge in terms of both safety and power consumption in interventional medical devices.

### 6.3. Relationship Between Photoacoustic Imaging and Challenges

As described in Section 4, CMUTs have demonstrated significant advantages in photoacoustic imaging applications, including their wide bandwidth, flexible design and fabrication capabilities, and optical transparency.

Researchers from Canon Inc. and Kyoto University have reported photoacoustic imaging systems specifically designed for human breast cancer diagnosis, utilizing CMUTs as the ultrasound receiver [115,117]. In these cases, the CMUTs act as the receiver to detect photoacoustic signals generated by laser excitation, thereby avoiding the performance limitations associated with lower output pressure that typically affect CMUTs in transmit mode. However, because photoacoustic imaging often requires long scanning times to acquire sufficient data for high-quality reconstruction, the charging effects in CMUTs become more significant when the device is maintained under high DC bias voltage. Therefore, it may lead to operating point shifts, potential instability in sensitivity, or introduction of artifacts over time.

Furthermore, in applications of photoacoustic and ultrasound dual-modal imaging, the lower output pressure of CMUTs in transmit mode remains a notable challenge. This limitation restricts imaging penetration depth, SNR, and overall image quality in the ultrasound component, thereby constraining the effectiveness of integrated dual-mode systems.

### 6.4. Interconnection Between the Challenges

The three challenges outlined in Section 5, limited output pressure, dielectric charging effects, and the requirement for high DC bias voltage, are not isolated issues and are closely interconnected. For example, to increase output pressure, the DC bias voltage is often set close to the pull-in voltage. This high electric field accelerates charge injection, worsening charging effects and causing operating point drift, which actually reduces output pressure. To compensate or restore performance, the bias voltage should be further increased. However, this additional increase in bias voltage intensifies the electric field even more, thereby aggravating the charging effects in a self-reinforcing cycle. Similarly, operating the CMUT in collapse mode to achieve higher output pressure also dramatically increases the electric field, making charging effects more severe and often requiring even higher bias to stabilize.

This strong interdependence implies that optimizing one parameter generally compromises the performance of the others. Such trade-offs not only make it difficult to achieve balanced improvements across all three aspects simultaneously but also tend to increase fabrication complexity and costs. As a result, for these challenges, systematic and overall solutions are required rather than isolated optimizations of individual parameters. Table 8 lists the effects of existing optimization strategies on output pressure improvement, charging effects, DC bias voltage, and fabrication complexity. As described in this table, a single strategy is often insufficient to effectively address all three challenges simultaneously.

Furthermore, as indicated in Table 6, modifications to some structural components of CMUT, such as membrane, electrodes, and cavity, can simultaneously enhance both output pressure and receive sensitivity. For example, the T-shaped cavity CMUT described in [138] was analyzed using FEM simulations, demonstrating improvements in both transmit and receive performance while also achieving a reduction in the pull-in voltage. Similarly, ref. [144] experimentally investigated a collapse-mode CMUT with an annular embossed pattern on the membrane, which resulted in increased output pressure and lower pull-in voltage.

From the perspective of operating mode, as shown in Table 6, collapse-mode CMUTs exhibit significant improvements in both transmit and receive performance. According to the 2021 European MEMS Ultrasound Benchmark technical white paper conducted by companies and research institutions, compared with conventional CMUTs, collapse-mode CMUTs achieved enhancements in bandwidth and peak pressure, enabling a superior balance of transmit and receive performance [169]. Although collapse-mode CMUTs require a DC bias voltage exceeding the pull-in voltage during operation that results in significant charging effects, references [156,167] indicate that by employing pre-charging methods to inject and store charges within CMUTs, zero-bias operation can be effectively achieved and sustained for 1.5–2.5 years. Furthermore, as indicated in Table 6, modifications to key structural components of the membrane, electrodes, and cavity can simultaneously enhance both output pressure and receive sensitivity. For example, the T-shaped cavity CMUTs in [138] demonstrated improvements in both transmit and receive performance while also achieving lower pull-in voltage. Similarly, ref. [144] investigated a collapse-mode CMUT cell with an annular embossed pattern designed on the membrane, which resulted in increased output pressure, fractional bandwidth, and lowered pull-in voltage.

Based on the above analysis, combining pre-charged collapse-mode CMUT design with structural optimization, such as specialized cavity shapes or nonuniform membranes, may offer a promising approach to address all three challenges concurrently. This combined strategy may leverage the advantages of collapse-mode operation while mitigating drawbacks such as high bias requirements and charging effects and further improving output pressure, sensitivity, and bandwidth. However, it should be noted that such combined modifications typically increase the complexity of the design and fabrication process, which may bring additional challenges in terms of manufacturing consistency, yield, and cost.

### 6.5. Future Outlook

First, ensuring the long-term stability of the injected charge in pre-charged collapse-mode CMUTs is a major challenge. Over time and under varying operating environments, such as interventional imaging or implantable devices, the charges stored in the insulating layer may leak or redistribute, leading to performance drift or even device failure. Therefore, developing industrial-grade reliability in charge engineering and packaging is critical. Furthermore, in pre-charged collapse-mode CMUTs, the central region of the membrane remains permanently collapsed. Maintaining the membrane’s mechanical strength and deformation stability under long-term and variable operating conditions requires further research on structural design and material selection.

Second, zero-bias CMUTs with high output pressure offer greater advantages for integration with low-voltage CMOS circuits. Fabricating CMUTs with pre-processed CMOS wafers can significantly reduce parasitic capacitance, improve receive SNR, lower power consumption, and enable on-chip beamforming and signal processing. This monolithic chip integration approach will promote further miniaturization and is highly suitable for developing compact ultrasound devices with low power consumption.

Third, pre-charged collapse-mode CMUTs have high output pressure and receive sensitivity without the need for a DC bias voltage. This significantly reduces system integration complexity and improves device safety. Therefore, this approach is suitable for high-frequency and high-resolution imaging in catheter-based interventional ultrasound devices, such as IVUS and ultrasound/photoacoustic dual-modal imaging systems.

Over the past three decades, driven by progress in MEMS technology and integrated circuits, CMUTs have successfully entered and been adopted in many medical imaging applications previously served by PZT ultrasound transducers. Nevertheless, the broader applications and future advancements of CMUTs remain constrained by various challenges, including the aforementioned issues and others. If the above goals can be achieved, pre-charged collapse-mode CMUTs are expected to extend to a broader range of scenarios, including implantable ultrasound monitoring, endoscopic photoacoustic imaging, and wearable ultrasound patches. These achievements will accelerate the clinical translation and commercial deployment of the next generation of lead-free high-performance ultrasound transducers.

## Figures and Tables

**Table 1:** Comparison of conventional surface micromachining and wafer-bonding process.

Features	Surface Micromachining	Wafer Bonding
Structural Flexibility	Lower, limited by film depositions and sacrificial layer relese.	Higher, diverse in structure.
Membrane Uniformity	Lower.	Higher.
Vacuum Packaging	Achieving a highly reliable vacuum seal is difficult.	Easily achievable, beneficial for enhancing performance and stability.
CMUTs Fill Factor	Lower fill factor due to etching holes.	Higher fill factor, no etch holes needed.
CMOS Integration	Compatible with CMOS technology.	Additional bonding steps are required.
Fabrication Costs	Relatively low, using MEMS standard processes.	Relatively high, wafer-bonding equipment is required.
Frequency Range	Moderate.	Wide.

**Table 2:** Comparison of CMUTs and piezoelectric transducers.

Features	CMUTs	Piezoelectric Transducers
Fractional Bandwidth	Generally exceed 100%.	Typically less than 80%.
Center Frequency Adjustability	Determined by the dimension and material of the membrane. Easily enable multi-frequency.	Determined by the thickness of the piezoelectric material. Challenges in multi-frequency.
Acoustic Impedance Matching	Approximately 2–3 MRayl, closer to soft tissues of the human body (1.5–1.6 MRayl).	Up to 30 MRayl (PZT), multilayer matching layers are required.
High-Density Array Capability	Easy to achieve high-density arrays.	Complex for high-density arrays.
CMOS Integration	Directly integrated into a single chip.	Requires wire bonding.
Output Pressure	Relatively low.	High, suitable for HIFU treatment.
Driving methods	DC bias voltage + AC voltage.	AC voltage.
Linearity	Medium, may be improved through appropriate bias control.	Good within a moderate dynamic range.
Self-heating	Low heat generation and high thermal conductivity.	Easy to self-heat and low thermal conductivity.
Thermal Performance	Minimal temperature dependence.	Temperature-sensitive with Curie temperature limitation.
Biocompatibility	Silicon-based materials have good biocompatibility.	Lead-based traditional materials; good after optimized encapsulation.

**Table 4:** Features of the Butterfly iQ series.

Item	Butterfly iQ	Butterfly iQ+	Butterfly iQ3
Frequency range	1–10 MHz	1–10 MHz	1–12 MHz
Probe type	Linear Convex Phased	Linear Convex Phased	Linear Convex Phased
Transducers	9000-element CMUTs	9000-element CMUTs	9000-element CMUTs
Imaging modes	B-mode, M-mode, Color Doppler, Power Doppler.	B-mode, M-mode, Color Doppler, Power Doppler, Biplane, Needle Viz Tool.	B-mode, M-mode, Color Doppler, Power Doppler Biplane, Needle Viz Tool, iQ Slice, iQ Fan.
Presets	19 clinical applications.	24 clinical applications.	25 clinical applications.
Price	USD 2999	USD 2999	USD 3899
Probe weight	313 g	309 g	300 g

**Table 5:** Features of transparent CMUTs.

Frequency	Bandwidth	Fabrication	Material	Transparency
5 MHz [95]	116%	Surface micromachining	Si, Cr/Au, polysilicon	Near-infrared
4.75 MHz (air) [108]	N/A	Anodic bonding	Glass, ITO, Si	50% @ 700–1000 nm
1.4 MHz [109]	105%	Anodic bonding	Glass, ITO, Si, Si_3_N_4_	30–40% @700–800 nm; 40–60%@ 800–900 nm
2 MHz [110]	52.3%	Adhesive wafer bonding	Glass, ITO, BCB, Si_3_N_4_	>70% @ 504–605 nm
8 MHz [111]	75%	Adhesive wafer bonding	Glass, ITO, BCB, Si_3_N_4_	average >70% @ 560–1000 nm
9 MHz [35]	150%	Adhesive wafer bonding	Fused-silica, ITO, BCB, Si_3_N_4_	Up to 90% in the visible light range.
4.2 MHz, 9.3 MHz [104]	86%, 77%	Adhesive wafer bonding	Fused-silica, ITO, BCB, Si_3_N_4_	Up to 76.8% in the visible light range.
3.5 MHz [112]	80%	Adhesive wafer bonding	PDMS, Fused-silica, ITO, BCB, Si_3_N_4_	Up to 67% in the visible light range.

**Table 6:** Methods for improving the output pressure of CMUTs.

Item	Method	Tx Improv.	Rx Improv.	Freq.	Ref.
Unconventional operating mode	Collapse-snapback	83.3%	N/A *	4.2 MHz	[119]
	Collapse	59.5%	better	20–28 MHz	[121]
	Collapse	94%	77%	2.3 MHz	[122]
	Collapse	107.9%	N/A	10 MHz	[123]
	Deep-collapse	Better	N/A	6.8 MHz	[124]
Unconventional membrane	Center-mass	82.5%	95.0%	2.5 MHz	[126]
	Center-mass	23.4%	N/A	3.6 MHz	[128]
	Trenches	122.2%	N/A	6.9 MHz	[129]
	Substrate-embedded springs	N/A	N/A	1.85 MHz	[130]
Unconventional electrodes	Dual-top	118.0%	13%	9 MHz	[132]
	Dual-top	142.4%	180%	8 MHz	[133]
	Dual-bottom	91.7%	9.3%	914 kHz	[136]
	Annular electrode	255% (air)	reduced	3 MHz	[137]
Unconventional cavity	T-shape	56.1% (air)	62.6% (air)	1.5 MHz	[138]
	2-stage-Optimal	N/A	N/A	0.9 MHz	[139]
Unconventional membrane–electrodes	Annular electrode membrane groove	37%	N/A	1.6 MHz	[140]
	Indirectly clamped CMUT	72%	N/A	4.85 MHz	[141]
	Dual-layer CMUT	17.7%	101.6%	0.25 MHz	[142]
Unconventional mode-membrane	Embossed + collapse-mode CMUT	88.1%	N/A	3.73 MHz	[143]
	Embossed + collapse-mode CMUT	27.1%	N/A	6.1 MHz	[144]

* Not available.

**Table 7:** Comparison of methods for reducing charging effects.

Methods	Strategy	Advantages	Challenges
Material Optimization	High-k dielectric insulation layer with annealing and SiC membrane structure.	Improving reliability from the fundamentals of materials.	Material compatibility in fabrication increases fabrication complexity.
Structure Optimization	Altering the membrane/electrode structure, adding posts inside the cavity.	Simultaneously improve output pressure, sensitivity, and bandwidth.	High design and manufacturing difficulty and complexity.
Driver Optimization	Adjusting bias voltage or excitation voltage.	No modifications to CMUT structure or materials required.	Requires complex drive circuitry and increases power consumption.
Pre-charged Method	Actively injects and stores charge in a floating electrode or storage layer.	Low power consumption and no bias needed.	Charge injection efficiency and stability.

**Table 8:** Impacts of optimization strategies on the three challenges.

Strategy	Pressure Improvement	Charging Effects	DC Bias	Fabrication Complexity
High-k materials	Low.	Low.	Reduced.	Moderate.
Structure optimization	Moderate to high.	Moderate.	Reduced.	Complex.
Collapse mode	High.	Significant.	High.	Low.
Pre-charged	Low.	Low.	Eliminated or reduced.	Complex.
Bias/driver optimization	Low to moderate.	Low.	Bipolar, or compensated.	Low.

## Data Availability

No new data were created or analyzed in this study.

## Abbreviations

The following abbreviations are used in this manuscript:

CMUTs	Capacitive micromachined ultrasonic transducers
PZT	Lead zirconate titanate
PMN-PT	Lead magnesium niobate–lead iitanate
KNN	Potassium sodium niobate
PVDF	Polyvinylidene fluoride
MEMS	Microelectromechanical systems
MUTs	Micromachined ultrasonic transducers
CMOS	Complementary metal-oxide semiconductor
PMUTs	Piezoelectric micromachined ultrasonic transducers
PolyMUMPs	Polysilicon Multi-User MEMS Processes
PI	Polyimide
SOI	Silicon-on-insulator
LOCOS	Localized oxidation of silicon
BCB	Benzocyclobutene
LTCC	Low-temperature co-fired ceramics
LPCVD	Low-pressure chemical vapor deposition
PECVD	Plasma-enhanced chemical vapor deposition
ITO	Indium tin oxide
AFE	Analog front-end
SNR	Signal-to-noise ratio
HIFU	High-intensity focused ultrasound
IVUS	Intravascular ultrasound
ICE	Intracardiac echocardiography
POCUS	Point-of-care ultrasound
SoC	System on chip
IJV	Internal jugular vein
RAP	Right atrial pressure
AHF	Acute heart failure
LUS	Lung ultrasound
PACT	Photoacoustic computed tomography
PAM	Photoacoustic microscopy
PDMS	Polydimethylsiloxane
FEM	Finite element method
EP	Electrode posts
IIP	Isolation insulating posts
